# Insulin-like growth factor 1-induced enolase 2 deacetylation by HDAC3 promotes metastasis of pancreatic cancer

**DOI:** 10.1038/s41392-020-0146-6

**Published:** 2020-05-13

**Authors:** Yan Zheng, Chao Wu, Jimeng Yang, Yue Zhao, Huliang Jia, Min Xue, Da Xu, Feng Yang, Deliang Fu, Chaoqun Wang, Beiyuan Hu, Ze Zhang, Tianen Li, Shican Yan, Xuan Wang, Peter J. Nelson, Christiane Bruns, Lunxiu Qin, Qiongzhu Dong

**Affiliations:** 1 Department of General Surgery, Huashan Hospital & Cancer Metastasis Institute & Institutes of Biomedical Sciences, Fudan University, Shanghai, 200040 China; 2 General, Visceral and Cancer Surgery, University Hospital of Cologne, Cologne, 50937 Germany; 3 Department of Pancreatic Surgery, Pancreatic Disease Institute, Huashan Hospital, Shanghai Medical College, Fudan University, Shanghai, China; 4 Medical Clinic and Policlinic IV, Ludwig-Maximilian-University (LMU), Munich, Germany

**Keywords:** Cancer metabolism, Metastasis, Cancer metabolism, Metastasis

## Abstract

Enolase 2 (ENO2) is a key glycolytic enzyme in the metabolic process of glycolysis, but its potential function in pancreatic ductal adenocarcinoma (PDAC) is unclear. In this study, we observed a significant overexpression of ENO2 in PDAC tissues, and its expression was correlated with metastasis and poor prognosis in PDAC patients. K394 was identified as a major acetylation site in ENO2 that regulates its enzymatic activity, cell metabolism and PDAC progression. Knockdown of ENO2 suppressed tumor growth and liver metastasis in PDAC. Re-expression of wild-type (WT) ENO2, but not the K394 acetylation mimetic mutant, could reverse the decreased tumor malignancy. We further characterized histone deacetylase 3 (HDAC3) and P300/CBP-associated factor (PCAF) as the potential deacetylase and acetyltransferase for ENO2, respectively. HDAC3-mediated deacetylation was shown to lead to ENO2 activation and enhancement of glycolysis. Importantly, insulin-like growth factor-1 (IGF-1) was found to decrease K394 acetylation and stimulate ENO2 activity in a dose- and time-dependent manner. The PI3K/AKT/mTOR pathway facilitated the phosphorylation of HDAC3 on S424, which promoted K394 deacetylation and activation of ENO2. Linsitinib, an oral small-molecule inhibitor of IGF-1R, could inhibit IGF-1-induced ENO2 deacetylation by HDAC3 and the PI3K/AKT/mTOR pathway. Furthermore, linsitinib showed a different effect on the growth and metastasis of PDAC depending on the overexpression of WT versus K394-mutant ENO2. Our results reveal a novel mechanism by which acetylation negatively regulates ENO2 activity in the metastasis of PDAC by modulating glycolysis. Blockade of IGF-1-induced ENO2 deacetylation represents a promising strategy to prevent the development of PDAC.

## Introduction

Pancreatic cancer is one of the most rapidly lethal cancers. Recent data show that the 5-year survival of patients with pancreatic carcinoma remains as low as 5–15% worldwide.^1,2^ To date, pancreatic ductal adenocarcinoma (PDAC) is the most common subtype of pancreatic carcinoma, accounting for 85% of all pancreatic neoplasms^3^ and presenting with a very poor prognosis. With the development of new therapeutic strategies, the mortality rate of some common malignancies is decreasing, but the mortality of PDAC is still high.^1^ One of the major hallmarks of PDAC is metastasis. Over 50% of patients are diagnosed with local lymph node or distant organ metastasis, and they lose the opportunity for surgical treatment.^4^ Therefore, deciphering the mechanisms underlying PDAC metastasis may allow early intervention and improve the prognosis of this disease.

Recent studies have reported that alterations in cell metabolism contribute to malignant transformation and cancer progression.^5^ The dysregulation of cellular metabolism is now recognized as a core feature of cancer cells. In general, cancer cells exhibit a unique aerobic glycolysis phenotype for more metabolic substrate acquisition during tumorigenesis and metastasis (Warburg effect). Previous work from our group identified a set of altered metabolic genes and metabolites associated with liver cancer progression.^6^ PDAC cells are well adapted to grow in the context of severe metabolic stress, as the hypovascular, fibrotic tumor microenvironment results in extreme hypoxia and limited nutrient availability.^7^ Extensively reprogrammed metabolism is seen in PDAC, which helps foster the different energetic and biosynthetic demands seen.^8^ A recent study elucidated that glucose metabolism is involved in pancreatic tumors and that inhibition of glycogen synthase kinase 3 beta retards cancer progression in mice.^9^ Alterations in metabolic protein expression and associated post-translational modification (PTM) effects may underlie the necessary adaptive changes seen in cancer cell metabolism.^10^

Lysine acetylation (Kac) is an important reversible and dynamic protein PTM. A recent study reported that acetylation of the glycolytic enzyme phosphoglycerate kinase 1 could enhance the malignancy of liver cancer cells.^11^ Similarly, acetylation of 6-phosphofructo-2-kinase/fructose-2,6-bisphosphatase 3 was found to lead to cytoplasmic accumulation of this enzyme, which helped cells avoid apoptosis by enhancing glycolysis.^12^ Notably, this general phenomenon is not only associated with glucose metabolism. The majority of intermediate metabolic enzymes in lipid and glutamine metabolism have also been found to undergo acetylation.^13^ Thus, the acetylation status can affect enzyme activity and cellular metabolism. The dysregulation of lysine acetylation may contribute to diverse pathological conditions, such as cancers.^11,12,14^

In the glycolytic penultimate step, enolase 2 (ENO2), also called neuro-specific enolase (NSE), is responsible for the conversion of 2-phosphoglycerate (2-PGA) to phosphoenolpyruvate (PEP). ENO2 is a well-established tumor biomarker for cancers such as neuroendocrine tumors, prostate cancer, small-cell lung cancer, and metastatic neuroblastoma and the microvascular invasion status of liver cancer.^15^ In our previous gene expression profiling study, we found that ENO2 was one of the leading genes associated with the metastasis of hepatocellular carcinoma (HCC).^16^ To our knowledge, the relationship between ENO2 and PDAC has not been studied. Moreover, previous studies reported that ENO2 might be acetylated.^17,18^ Thus, a better understanding of the potential role of ENO2 acetylation in maintaining the malignant phenotype of PDAC cells may help identify novel potential targets for enhancing the efficacy of cancer therapeutics.

Here, we demonstrate that ENO2 expression correlates with metastasis and poor prognosis in PDAC. ENO2 is deacetylated at the K394 site in PDAC, and deacetylation of ENO2 is correlated with metastasis. Histone deacetylase 3 (HDAC3)-mediated ENO2 deacetylation plays a key role in regulating ENO2 activity/function upon insulin-like growth factor-1 (IGF-1) stimulation in PDAC. Linsitinib, an oral small-molecule inhibitor of IGF-1R, could inhibit IGF-1-induced ENO2 deacetylation. PDAC expressing wild-type (WT) but not K394-mutant ENO2 was sensitive to linsitinib. Our findings indicate that ENO2 deacetylation (at K394) is an important driver of PDAC malignancy and represents a useful biomarker for IGF-1R inhibitor responsiveness and metastasis of PDAC.

## Results

### ENO2 correlates with metastasis and prognosis of PDAC

**Fig. 1:**
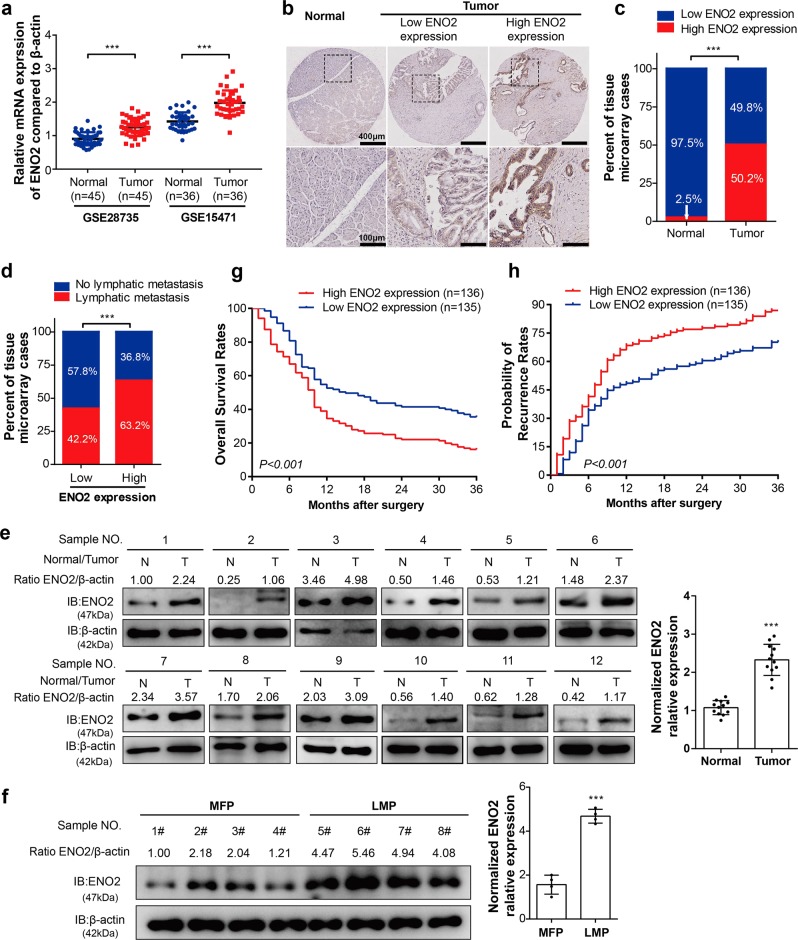
High ENO2 expression is closely associated with poor outcomes of patients with PDAC. **a** ENO2 mRNA levels in pancreatic tumor tissues and corresponding adjacent normal tissues in two independent GEO datasets (GSE28735, *n* = 45; GSE15471, *n* = 36). **b** Representative IHC staining results of ENO2: left panel: normal tissue; middle panel: low ENO2 expression; right panel: high ENO2 expression; scale bar = 400 μm or 100 μm. **c** High or low expression of ENO2 in tumor (*n* = 271) or adjacent normal tissues (*n* = 161) in the TMA cohort. **d** Existence or absence of lymph node metastasis in the low or high ENO2 expression group in the TMA cohort (ENO2 high, *n* = 136; ENO2 low, *n* = 135). **e**, **f** The protein levels of ENO2 in PDAC samples. Twelve pairs of tumor (T) and adjacent normal tissues (N) (*n* = 12 per group) (**e**) and eight tumor samples with or without liver metastasis (**f**) were analyzed by western blot with ENO2 antibody. Relative ENO2 protein levels were normalized against β-actin. MFPs, metastasis-free patients (*n* = 4); LMPs, liver metastasis patients (*n* = 4). **g** Overall survival rates in PDAC patients with low (*n* = 135) or high (*n* = 136) ENO2 expression assessed by Kaplan–Meier analysis. **h** Probability of tumor recurrence rates in PDAC patients with low (*n* = 135) or high (*n* = 136) ENO2 expression assessed by Kaplan–Meier analysis. Error bars represent the mean ± SD, and the dots represent the value of each experiment; ***P < 0.001. A paired *t* test was employed in (**a**) and (**e**), an unpaired *t* test was employed in (**f**), Fisher exact test was employed in (**c**), the chi-square test was employed in (**d**), and the log-rank test was employed in (**g**) and (**h**)

To investigate the possible role of ENO2 in pancreatic cancer progression, we first analyzed ENO2 mRNA levels based on two Gene Expression Omnibus (GEO) datasets (GSE28735 and GSE15471). Overall, ENO2 mRNA was found to be significantly increased in pancreatic cancer tissues compared with normal tissues (Fig. 1a, *P* < 0.001). To confirm these findings from published microarray datasets, immunohistochemistry (IHC) staining was used to assess the protein levels of ENO2 in 271 human PDAC tumors and 161 normal tissues from our hospital. Similar to what was found in public data, the protein level of ENO2 was also significantly increased in PDAC tissues compared with normal tissues in the cohort from our hospital (Fig. 1b, c). Interestingly, a significant difference in ENO2 expression was also observed between PDAC with lymphatic metastasis and metastasis-free tumors (Fig. 1d, *P* < 0.001). To further confirm the mRNA and IHC findings, we used immunoblotting to assess the expression levels of ENO2 in PDAC tissues from 12 patients. ENO2 protein levels were confirmed to be significantly higher in the PDAC tissues than in their matched normal tissues (Fig. 1e). Furthermore, in comparison with that in nonmetastatic PDAC tissues, the protein level of ENO2 was found to be increased in PDAC tissues with liver metastasis (Fig. 1f), suggesting that ENO2 may be involved in PDAC progression.

In addition, higher ENO2 expression levels also correlated with poor overall survival rates (OS) and an increased incidence of recurrence compared with low ENO2 expression levels (Fig. 1g, h). To better characterize the potential association between ENO2 expression and the prognosis of PDAC patients, the general correlation between ENO2 IHC staining in PDAC samples and patient clinicopathological features and prognosis after surgery was evaluated. ENO2 levels in tumor tissues were found to be significantly associated with tumor differentiation (*P* = 0.004) and lymph node metastasis (*P* < 0.001, Supplementary Table S1). Using univariate analysis, ENO2 expression, sex, CA19-9 level and tumor differentiation were shown to be significantly associated with OS and the probability of recurrence rate (PRR). In multivariate analyses, the prognostic value of ENO2 for OS and the PRR was independent of all other clinical variables tested (Supplementary Table S2). Collectively, these results suggest that high ENO2 expression is associated with poorer prognosis and worse malignancy than low ENO2 expression in PDAC.

### ENO2 is acetylated at K394 in PDAC

**Fig. 2:**
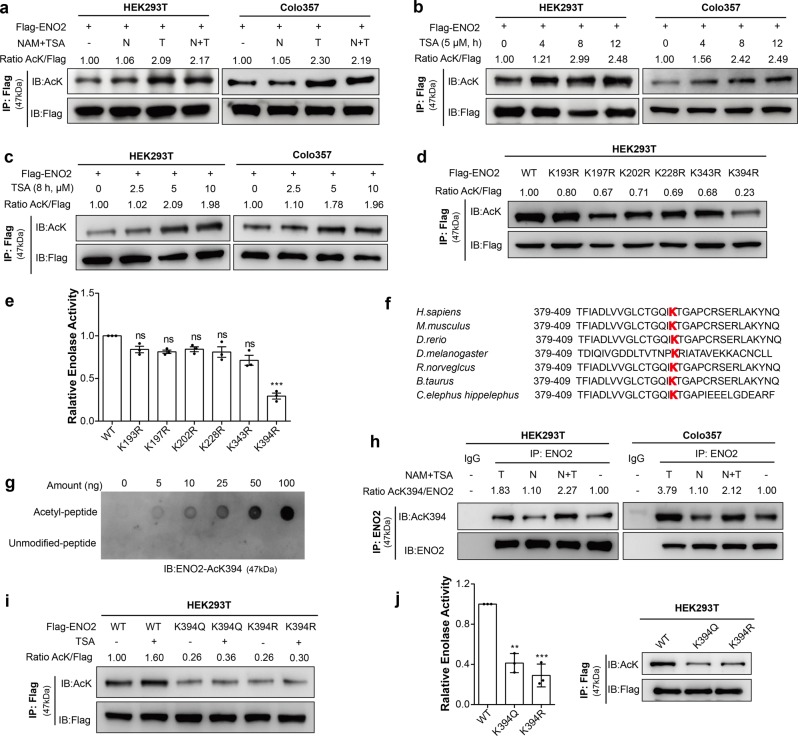
Acetylation of ENO2 at K394 decreases its enzymatic activity. **a** Flag-ENO2 was ectopically expressed in HEK293T and Colo357 cells, followed by treatment with NAM (nicotinamide, 10 mM for 6 h), TSA (trichostatin, 5 μM for 12 h), or both (N + T: nicotinamide, 10 mM for 6 h and trichostatin, 5 μM for 12 h). Acetylation levels of immunopurified Flag-ENO2 were examined by western blot analysis with a pan-acetylated lysine antibody (AcK). Relative ENO2 acetylation levels were normalized by Flag. **b**, **c** ENO2 acetylation levels were increased by TSA treatment in a time- (**b**) and dose- (**c**) dependent manner. HEK293T and Colo357 cells overexpressing Flag-ENO2 were treated with 5 μM TSA for 0, 4, 8, or 12 h or with 0 μM, 2.5 μM, 5 μM or 10 μM TSA for 8 h. ENO2 acetylation was determined by western blot with AcK. **d**, **e** Mapping the major regulatory sites of acetylation in ENO2. Flag-tagged WT ENO2 and the K193R, K197R, K202R, K228R, K343R, and K394R mutants were each overexpressed in HEK293T cells. Flag-ENO2 was immunoprecipitated, and its acetylation was examined with AcK, normalized against Flag (**d**). ENO2 activity was determined and normalized to protein levels (*n* = 3 per group). WT ENO2 activity was set as 1 (**e**). **f** K394 in ENO2 is evolutionarily conserved. The sequences around ENO2 K394 from different species are shown. **g** Characterization of the anti-acetyl-ENO2 (AcK394) antibody. Different amounts of acetyl-K394 peptide or unmodified peptide were spotted on nitrocellulose membranes, and the specificity of the antibody against the acetylated K394 residue was determined by dot blot assay. **h** Endogenous ENO2 protein was immunoprecipitated with anti-ENO2 antibody from HEK293T and Colo357 cells treated with NAM and TSA as indicated (NAM, 10 mM for 6 h; TSA, 5 μM for 12 h; or both NAM and TSA treatment). Acetylation levels of K394 were analyzed with an antibody specifically recognizing the acetylated ENO2 K394 residue (AcK394). Relative K394 acetylation levels were normalized against ENO2 protein. **i**, **j** K394 is the major acetylation residue of ENO2. Flag-tagged wild-type ENO2 and the K394Q and K394R mutants were each overexpressed in HEK293T cells, followed by treatments with or without 5 μM TSA for 8 h. The acetylation levels (**i**) and enzyme activity (**j**) of ENO2 were analyzed. The group with WT ENO2 served as the control group; *n* = 3 per group. Error bars represent the mean ± SD, and the dots represent the value of each experiment; **P < 0.01, ***P < 0.001, ns: no significance. Statistical significance was determined by unpaired *t* test

Previous proteomic studies have shown that ENO2 is acetylated on multiple lysine residues.^17,18^ To confirm this acetylation, Flag-tagged ENO2 was ectopically expressed in HEK293T (Fig. 2a, left) and pancreatic cancer cells (Fig. 2a, right), and the acetylation level was tested using a pan-anti-acetylated lysine antibody. The results demonstrated that ectopically expressed ENO2 was indeed acetylated and that its acetylation was significantly increased after treatment with trichostatin A (TSA), an inhibitor of HDACs, but not after treatment with nicotinamide (NAM), an inhibitor of the SIRT family deacetylases. Furthermore, the acetylation level of ENO2 was increased by TSA treatment in a time- (Fig. 2b) and dose-dependent (Fig. 2c) manner, suggesting that ENO2 is acetylated in PDAC.

After confirming that ENO2 was acetylated, we then sought to identify which residue in ENO2 represented the functional acetylation regulatory site. Among the six potential sites identified, two of the lysine residues (K343 and K394) are located in the active center of ENO2, while the other four (K193, K197, K202, and K228) have been previously described.^17,18^ To determine which lysine residue(s) plays a major role in the regulation of ENO2, each of the acetylated lysine residues in ENO2 was mutated to arginine (R), and the acetylation level and enzyme activity were evaluated individually. Among the sites identified, substitution at K394, but not at the other five lysine residues, substantially reduced ENO2 acetylation (Fig. 2d) and enzyme activity (Fig. 2e), indicating that K394 plays an important role in controlling ENO2 activity. In addition, K394 was found to be evolutionarily conserved across several different species (Fig. 2f). To further characterize the K394 acetylation site, an antibody (AcK394-ENO2) was generated that specifically recognizes ENO2 when it is acetylated at the K394 site (Supplementary Fig. S1a). Dot blot assays showed that the AcK394 antibody preferentially detected the acetylated peptide but not the unmodified peptide, demonstrating the specificity of this antibody (Fig. 2g). K394 acetylation was further verified by immunoprecipitation (IP) of endogenous ENO2 in HEK293T and pancreatic cancer cells (Fig. 2h). Importantly, the K394 acetylation level of ENO2 could be increased by treatment with TSA. However, both the K394R and K394Q mutants exhibited a negligible change in acetylation levels upon TSA treatment (Fig. 2i). Because ENO2 is an important glycolytic enzyme contributing to cancer cell energetics, we hypothesized that K394 acetylation may modulate ENO2 enzymatic activity. As expected, both the K394R and K394Q mutants exhibited much lower activity than WT ENO2 (Fig. 2j), reaffirming that K394 is a major acetylation site in ENO2.

### ENO2 K394 deacetylation is crucial for PDAC glycolysis and metastasis

**Fig. 3:**
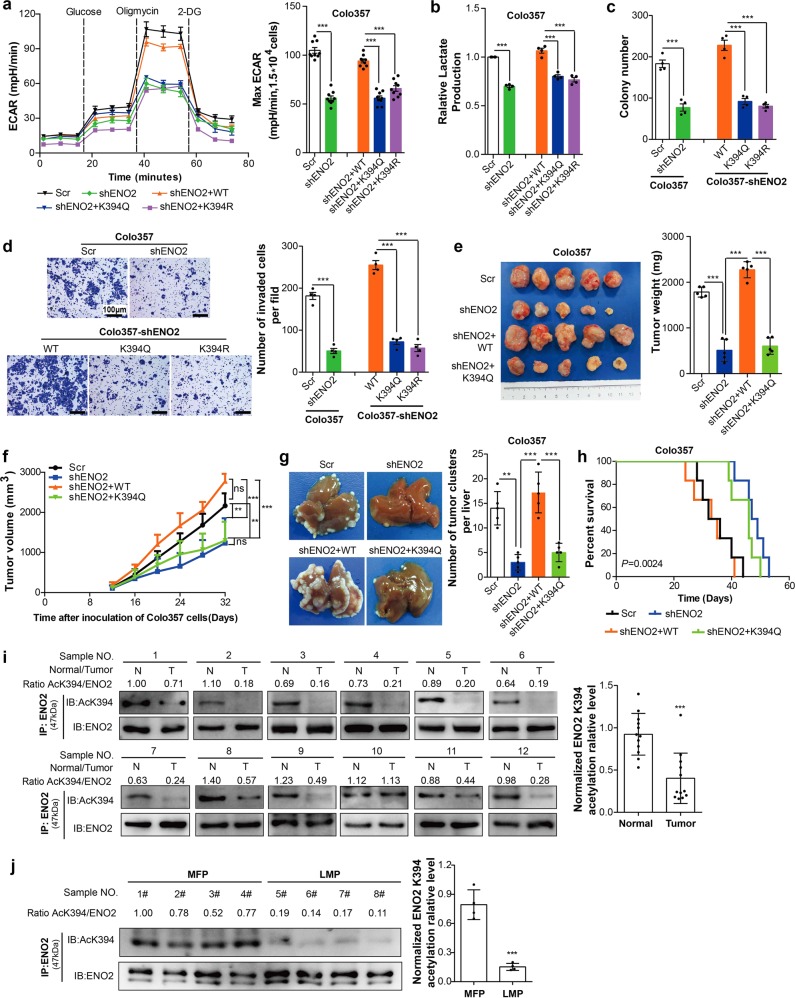
ENO2 K394 acetylation represses glycolysis and invasion of PDAC cells in vitro and in vivo. **a** WT ENO2 and its K394R and K394Q mutants were reintroduced into ENO2 knockdown Colo357 cells. The extracellular acidification rate (ECAR) of the indicated cells was detected by using a Seahorse XF96 Extracellular Flux Analyzer, and the maximum glycolytic rates were summarized, *n* = 9 per group. **b** Lactate secretion in the culture medium of the indicated cells for 12 h; *n* = 4 per group. **c**, **d** Colony assays (**c**) and transwell assays (**d**) were performed with the indicated Colo357 cells; *n* = 4 per group (scale bar = 100 μm). **e**, **f** Colo357 cells with ENO2 knockdown and Colo357 cells with ENO2 knockdown plus reintroduced with wild-type ENO2 or K394Q mutants were injected into the flanks of nude mice (*n* = 5 for each group). The tumor volumes (**e**, left), tumor weight (**e**, right) and tumor growth curves (**f**) of subcutaneous implantation models of PDAC at day 32 are shown. **g**, **h** The effect of ENO2 K394 acetylation on metastasis of PDAC in metastatic mouse models (*n* = 5 per group). **g** Livers were harvested after mice died, and metastatic nodules were counted by H&E staining (See Supplementary Fig. S2e). The total numbers of liver metastatic lesions were summarized. **h** Kaplan–Meier survival curve of mice after intrasplenic injection of Colo357 cells with ENO2 knockdown and Colo357 cells with ENO2 knockdown plus reintroduced with wild-type ENO2 or K394Q mutants. **i**, **j** The K394 acetylation levels of ENO2 in PDAC samples. Twelve pairs of tumor (T) and adjacent normal tissues (N) (*n* = 12 per group) (**i**) and eight tumor samples with or without liver metastasis (**j**) were subjected to IP with ENO2 antibody and western blot to detect K394 acetylation of ENO2. MFPs, metastasis-free patients (*n* = 4); LMPs, liver metastasis patients (*n* = 4). Error bars represent the mean ± SD, and the dots represent the value of each experiment; **P < 0.01, ***P < 0.001, ns: no significance. An unpaired *t* test was employed in (**a**–**d, j**), one-way ANOVA followed by Bonferroni’s post hoc test was employed in (**e**) and (**g**), two-way ANOVA followed by Bonferroni’s post hoc test was employed in (**f**), the log-rank test was employed in (**h**), and a paired *t* test was employed in (**i**)

To address the functional significance of ENO2 regulation by K394 acetylation, we generated stable PDAC cells in which endogenous ENO2 was depleted, and WT or K394-mutant ENO2 was reintroduced (Supplementary Fig. S1b, c). Because ENO2 is a major metabolic enzyme in the glycolysis pathway, we used extracellular acidification measurements to determine the potential changes in metabolism after ENO2 K394 acetylation. Depletion of endogenous ENO2 decreased the extracellular acidification rate (ECAR) of cells to suppress glycolysis, which was effectively restored by re-expression of WT ENO2 but not with the K394 mutants (Fig. 3a and Supplementary Fig. S2a, b). Similar results were observed in tests of lactate production that were performed with the resulting cell lines (Fig. 3b). These findings strongly suggested that ENO2 K394 acetylation represented an essential step in glycolytic metabolism in cancer cells.

Cancer cells depend on enhanced glycolysis to maintain their rapid growth. We hypothesized that K394 acetylation of ENO2 is associated with tumor cell progression. As expected, knockdown of ENO2 resulted in significant suppression of colony formation and invasion. However, the re-expression of WT ENO2, but not of the K394-mutant, reversed the growth retardation phenotype of PDAC cells caused by ENO2 depletion (Fig. 3c, d and Supplementary Fig. S2c, d), suggesting an important function of K394-acetylated ENO2 in vitro.

To test the function of ENO2 K394 acetylation in vivo, PDAC cells stably expressing Scr, shENO2, shENO2-ENO2 WT or shENO2-ENO2 K394Q were implanted into nude mice to establish a subcutaneous mouse model. Knockdown of ENO2 significantly decreased tumor growth and tumor volume; however, the tumor growth inhibition was reversed by WT ENO2 but not by the K394-mutant (Fig. 3e, f). To further characterize the effects of ENO2 K394 acetylation on tumor metastasis, we developed in vivo metastatic mouse models. Compared with the respective values in the Scr group, the number of metastatic nodules in livers was found to decrease and survival was prolonged in the shENO2 group. More importantly, WT ENO2, but not the K394-mutant, was found to significantly promote in vivo liver metastases and shorten survival in mice (Fig. 3g, h and Supplementary Fig. S2e).

To characterize the potential clinical relevance of ENO2 K394 acetylation, ENO2 K394 acetylation levels were examined in PDAC samples. Immunoblotting of the PDAC samples showed that ENO2 K394 acetylation levels were significantly lower in the PDAC tissues than in their matched normal tissues (Fig. 3i). Furthermore, in comparison to those in nonmetastatic PDAC tissues, ENO2 K394 acetylation levels were significantly lower in PDAC tissues with liver metastasis (Fig. 3j). Next, we analyzed ENO2 K394 acetylation in a panel of PDAC cell lines and human pancreatic duct epithelial (HPDE) cells. ENO2 K394 acetylation levels were significantly decreased in six established PDAC cell lines relative to those in the nontransformed pancreatic cell line HPDE. In addition, ENO2 K394 acetylation levels in L3.6pl cells with high potential for liver metastasis were much lower than those found in the other low-metastatic PDAC cell lines (Supplementary Fig. S2f). Therefore, ENO2 K394 deacetylation plays important roles in promoting PDAC growth and metastasis.

### PCAF is a potential acetyltransferase of ENO2

**Fig. 4:**
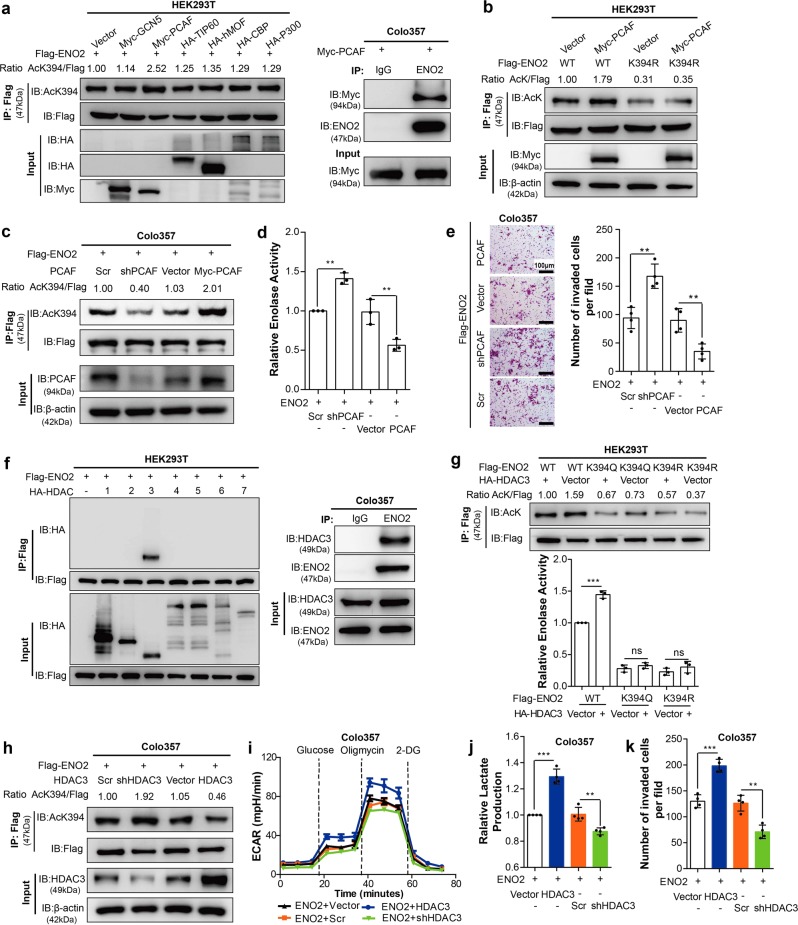
PCAF and HDAC3 regulate ENO2 K394 acetylation. **a** PCAF regulates ENO2 acetylation. HEK293T cells were transfected with empty vector, Myc-tagged GCN5/PCAF, HA-tagged TIP60/hMOF/CBP/P300 or Flag-tagged ENO2 for 48 h. ENO2 was immunopurified with anti-Flag beads, and K394 acetylation was detected by western blot and normalized against Flag (left). The interaction between Myc-tagged PCAF and endogenous ENO2 was detected by western blot (right). **b** PCAF acetylates ENO2 at K394. Flag-tagged wild-type ENO2 or its K394R mutant was coexpressed with empty vector or Myc-tagged PCAF in HEK293T cells, followed by TSA (5 μM) treatment for 12 h. ENO2 acetylation levels were determined with an anti-AcK antibody and normalized by Flag. **c–e** PCAF increases the acetylation of ENO2 and inhibits its activity. Knockdown or overexpression of Myc-tagged PCAF in Colo357 cells with ENO2 stably overexpressed was followed by TSA (5 μM) treatment for 12 h. ENO2 K394 acetylation levels (**c**), ENO2 enzyme activity (**d**, *n* = 3 per group) and invasive ability (**e**, *n* = 4 per group) in Colo357 cells were determined (scale bar = 100 μm). **f** ENO2 interacts with HDAC3. Flag-tagged ENO2 was coexpressed with HA-tagged HDAC as indicated in HEK293T cells. The interaction between ENO2 and HDACs was determined by IP and western blot with anti-Flag and anti-HA antibodies (left). In Colo357 cells, ENO2 protein was immunopurified with ENO2 antibody followed by western blot to detect its interaction with endogenous HDAC3 (right). **g** HDAC3 decreases ENO2 K394 acetylation and activates its activity. Flag-tagged wild-type ENO2 or its K394Q/R mutants were coexpressed with or without HA-tagged HDAC3 in HEK293T cells, followed by IP and western blot with Flag beads and anti-Flag/AcK antibody. ENO2 acetylation levels were normalized against Flag (the upper panel). Determination of the enzyme activity of ENO2 in the indicated HEK293T cells was performed by enzyme assay (the bottom panel, *n* = 3 per group). **h–k** HDAC3 promotes ENO2 K394 deacetylation and invasion of PDAC cells. Knockdown or overexpression of HDAC3 in Colo357 cells with Flag-tagged ENO2 stably overexpressed. ENO2 K394 acetylation levels were detected with anti-AcK394 antibody and normalized against Flag (**h**). ECAR (**i**, *n* = 9 per group), relative lactate production (**j**, *n* = 4 per group) and migration (**k**, *n* = 4 per group) were measured in Colo357 cells as indicated. Error bars represent the mean ± SD, and the dots represent the value of each experiment; **P < 0.01, ***P < 0.001, ns: no significance. Statistical significance was determined by unpaired *t* test

The established role of acetyltransferases and deacetylases in tumor biology led us to search for potential acetyltransferases and deacetylases responsible for ENO2 K394 acetylation. When cotransfected with ENO2, we found that P300/CBP-associated factor (PCAF), but not GCN5, Tip60, hMOF, CBP or P300, was associated with ENO2 and increased ENO2 K394 acetylation levels (Fig. 4a, left). The interaction of PCAF with endogenous ENO2 was further validated by IP in PDAC cells (Fig. 4a, right). Ectopically expressed PCAF promoted WT ENO2 acetylation but had almost no effect on acetylation of the K394-mutant (Fig. 4b). Furthermore, depletion of endogenous PCAF effectively decreased ENO2 K394 acetylation in PDAC cells, supporting a functional role of PCAF in ENO2 acetylation (Fig. 4c and Supplementary Fig. S3a, b). More importantly, depletion of endogenous PCAF effectively reduced the enzyme activity of ENO2 and suppressed invasion of PDAC cells, which could be rescued by upregulation of PCAF (Fig. 4d, e and Supplementary Fig. S3c). Collectively, these results indicate that PCAF is a potential acetyltransferase of ENO2.

### HDAC3 deacetylates and activates ENO2

As TSA was observed to be more potent than NAM for enhancing ENO2 acetylation (Fig. 2a), we then sought to identify which HDAC enzyme(s) mediates ENO2 deacetylation. To test this hypothesis, we first carried out co-IP experiments with ENO2 and several HDAC members and found that ENO2 interacted with HDAC3 but not the other six HDACs when ENO2 and each HDAC were coexpressed in HEK293T cells (Fig. 4f, left). The endogenous protein interaction between HDAC3 and ENO2 could be detected in PDAC cells (Fig. 4f, right). Interestingly, co-overexpression of HA-HDAC3 with Flag-ENO2 WT decreased the acetylation level of ENO2 and increased its enzyme activity. In contrast, co-overexpression of HA-HDAC3 with the K394R/Q mutants did not change the acetylation or enzyme activity of ENO2 (Fig. 4g). However, knockdown of HDAC3 increased the K394 acetylation level (Fig. 4h and Supplementary Fig. S3d, e). Furthermore, knockdown of HDAC3 effectively inhibited ECAR, lactate production and the invasion capacity of PDAC cells, which could be restored by upregulation of HDAC3 (Fig. 4i–k and Supplementary S3f). In addition, we detected the expression level of HDAC3 in human PDAC tissues and found that there was a strong correlation between HDAC3 levels and both ENO2 protein levels and ENO2 K394 acetylation levels (Supplementary Fig. S4a–c). In summary, HDAC3 is the major deacetylase of ENO2 and is responsible for the deacetylation of K394 in PDAC.

### The IGF-1/mTOR pathway regulates K394 deacetylation by S424 phosphorylation of HDAC3

**Fig. 5:**
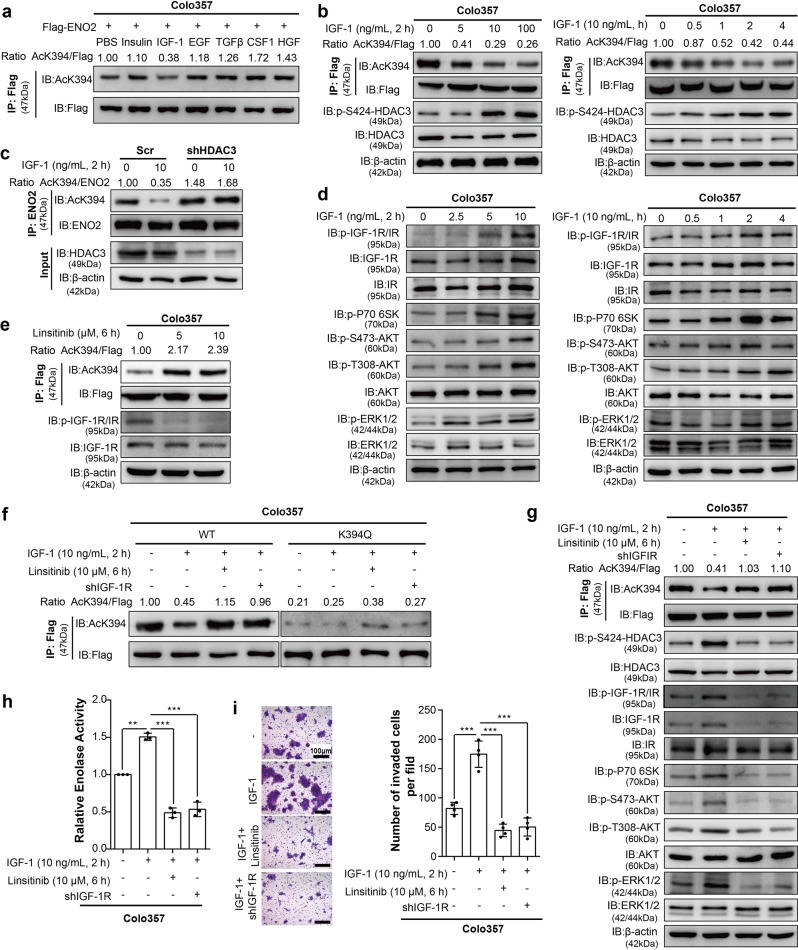
Inhibition of IGF-1R restrains the K394 deacetylation and enzymatic activation of ENO2-induced by IGF-1. **a** Flag-tagged ENO2 was stably overexpressed in Colo357 cells, followed by separate incubation with insulin (100 ng/mL, 12 h), IGF-1 (100 ng/mL, 12 h), EGF (30 ng/mL, 24 h), TGFβ (10 ng/mL, 72 h), CSF1 (10 ng/mL, 30 min) or HGF (50 ng/mL, 30 min) stimulation. ENO2 protein was immunopurified with Flag beads, and ENO2 K394 acetylation was analyzed by western blot with anti-AcK394 antibody. **b** IGF-1 induces ENO2 K394 deacetylation. Flag-tagged ENO2 was stably overexpressed in Colo357 cells treated with 0, 5, 10, or 100 ng/mL IGF-1 for 2 h (left) or 10 ng/mL IGF-1 for 0, 0.5, 1, 2, or 4 h (right). ENO2 was immunopurified with Flag beads, and K394 acetylation levels were determined by western blot and normalized against Flag. The protein levels of HDAC3 and HDAC3 S424 phosphorylation were detected. **c** HDAC3 knockdown inhibits the ENO2 K394 deacetylation induced by IGF-1. HDAC3 was stably knocked down in Colo357 cells following treatment with 0 or 10 ng/mL IGF-1 for 2 h. Immunoprecipitation and western blot were performed to validate the knockdown of HDAC3 and to detect the change in K394 acetylation levels. K394 acetylation levels were normalized against ENO2. **d** IGF-1 activates the PI3K/AKT/mTOR and ERK pathways. Colo357 cells treated with 0, 2.5, 5, or 10 ng/mL IGF-1 for 2 h (left) or treated with 10 ng/mL IGF-1 for 0, 0.5, 1, 2, or 4 h (right) were then harvested and subjected to western blot to evaluate the activation of the PI3K/AKT/mTOR and ERK pathways with the indicated antibodies. **e** Inhibition of IGF-1R increased ENO2 K394 acetylation. Colo357 cells stably overexpressing Flag-tagged ENO2 were treated with 0, 5, or 10 μM linsitinib for 6 h. The phosphorylation of IGF-1R and ENO2 K394 acetylation were assessed by IP and western blot using the indicated antibodies. **f** Linsitinib restored the K394 acetylation level in cells with wild-type ENO2 reintroduction but not in K394Q-mutant cells. Colo357 cells stably overexpressing Flag-tagged ENO2 were treated with IGF-1 (10 ng/mL, 2 h) stimulation, linsitinib (10 μM, 6 h) plus IGF-1 (10 ng/mL, 2 h) stimulation or IGF-1R knockdown plus IGF-1 (10 ng/mL, 2 h) stimulation. ENO2 proteins were immunoprecipitated, and K394 acetylation was examined with anti-AcK394 and normalized against Flag. **g** Inhibition of IGF-1R depressed the ENO2 K394 deacetylation and the activation of the PI3K/AKT/mTOR and ERK pathways induced by IGF-1. Colo357 cells treated as indicated in Fig. 5g were subjected to immunoprecipitation and western blot with the indicated antibodies to elucidate K394 acetylation levels (normalizing against Flag), the phosphorylation levels of HDAC3 S424 and the activity of the PI3K/AKT/mTOR and ERK pathways. **h–i** IGF-1R inhibition suppressed the enzymatic activity of ENO2 and invasion of tumor cells induced by IGF-1. Enzymatic activity assays (**h**, *n* = 3 per group) and transwell assays (**i**, *n* = 4 per group) were performed with the Colo357 cells indicated in Fig. 5g (scale bar = 100 μm). Error bars represent the mean ± SD, and the dots represent the value of each experiment; **P < 0.01, ***P < 0.001. Statistical significance was determined by one-way ANOVA followed by Bonferroni’s post hoc test

Previous studies have shown that the receptor tyrosine kinase (RTK) family plays critical roles in tumor progression and metabolism.^19^ Therefore, we hypothesized that some protein factors related to RTK may also act as regulators of ENO2 acetylation. When IGF-1, but not the other factors tested, was added to ENO2-overexpressing PDAC cell lines, ENO2 K394 acetylation was inhibited (Fig. 5a). Furthermore, IGF-1 treatment dose- and time-dependently decreased ENO2 K394 acetylation (Fig. 5b), suggesting that K394 is an important site for the ENO2 deacetylation induced by IGF-1. Then, we sought to investigate how IGF-1 regulates ENO2 K394 acetylation. The suppressive effect of IGF-1 on endogenous ENO2 K394 acetylation could be eliminated when HDAC3 was depleted (Fig. 5c).

Recent studies have identified HDAC3 as a protein that undergoes phosphorylation, with serine (S) 424 representing a key phosphorylation site.^20,21^ We found that IGF-1 treatment did not change HDAC3 expression but was able to enhance the endogenous S424 phosphorylation of HDAC3 in a time- and dose-dependent manner (Fig. 5b). Furthermore, co-overexpression of ENO2 and WT HDAC3 or the HDAC3 S424D mutant (which mimics phosphorylation^22^) could facilitate the deacetylation of WT ENO2 K394. However, the HA-HDAC3 S424A-mutant (which mimics dephosphorylation^23^) increased the K394 acetylation level of ENO2. In contrast, co-overexpression of HA-HDAC3 S424A or the S424D mutant with the ENO2 K394Q mutant did not change the acetylation of ENO2 (Supplementary Fig. S5a). In addition, IGF-1 treatment promoted the phosphorylation of HDAC3 S424 and its mediated ENO2 deacetylation when WT HDAC3 was ectopically expressed but had almost no effect on acetylation of K394 when S424A-mutant HDAC3 was ectopically expressed (Supplementary Fig. S5b). However, IGF-1 stimulation did not change the interaction of HDACs with ENO2 (Supplementary Fig. S5c). Furthermore, we checked HDAC3 phosphorylated S424 levels in PDAC samples and found that HDACpS424 was positively correlated with ENO2 protein expression and negatively correlated with ENO2 K394 acetylation (Supplementary Fig. S4a–c). Collectively, these data suggest that IGF-1 regulates ENO2 K394 acetylation by controlling HDAC3 S424 phosphorylation.

Recent studies have suggested a critical role of the mTOR signaling pathway in HDAC3 phosphorylation.^11^ In addition, the PI3K/AKT/mTOR pathway is an important pathway regulated by a variety of cellular signals, including IGF-1/IGF-1R.^24,25^ Therefore, we proposed that the PI3K/AKT/mTOR signaling pathway may regulate ENO2 K394 acetylation in response to IGF-1. To test this hypothesis, we then studied the potential role of the PI3K/AKT/mTOR pathway in IGF-1-mediated signaling regulating ENO2 K394 acetylation. Notably, IGF-1 activated the PI3K/AKT/mTOR pathway in a time- and dose-dependent manner (Fig. 5d). Moreover, both LY294002 (PI3K inhibitor) and rapamycin (mTOR inhibitor) suppressed IGF-1-induced HDAC3 phosphorylation and ENO2 deacetylation at K394 (Supplementary Fig. S6).

### Inhibition of IGF-1 increases K394 acetylation and suppresses ENO2 activity

To validate the function of the IGF-1/mTOR pathway in the regulation of ENO2 K394 acetylation, we treated PDAC cells with linsitinib, an oral small-molecule inhibitor of IGF-1R.^26,27^ Linsitinib dose dependently increased K394 acetylation (Fig. 5e and Supplementary Fig. S7a–c). In addition, inhibition of IGF-1R by linsitinib or shIGF-1-R rescued the inhibition of ENO2 K394 acetylation induced by IGF-1 treatment. However, K394Q mutants showed no change in acetylation levels when the cells were treated with IGF-1 or inhibited by IGF-1R (Fig. 5f and Supplementary Fig. S7d, e). Furthermore, linsitinib or shIGF-1-R effectively abolished the activation of the PI3K/AKT/mTOR pathway and HDAC3 S424 phosphorylation (Fig. 5g and Supplementary Fig. S7f). Treatment with linsitinib or shIGF-1-R significantly decreased the ENO2 activity, colony formation and invasive ability of PDAC cells in vitro, which were increased by IGF-1 (Fig. 5h, i and Supplementary Fig. S7g–i). Collectively, these data indicate that the IGF-1/mTOR pathway is linked to K394 deacetylation and ENO2 activation.

### PDAC with ENO2 K394 deacetylation is sensitive to linsitinib

**Fig. 6:**
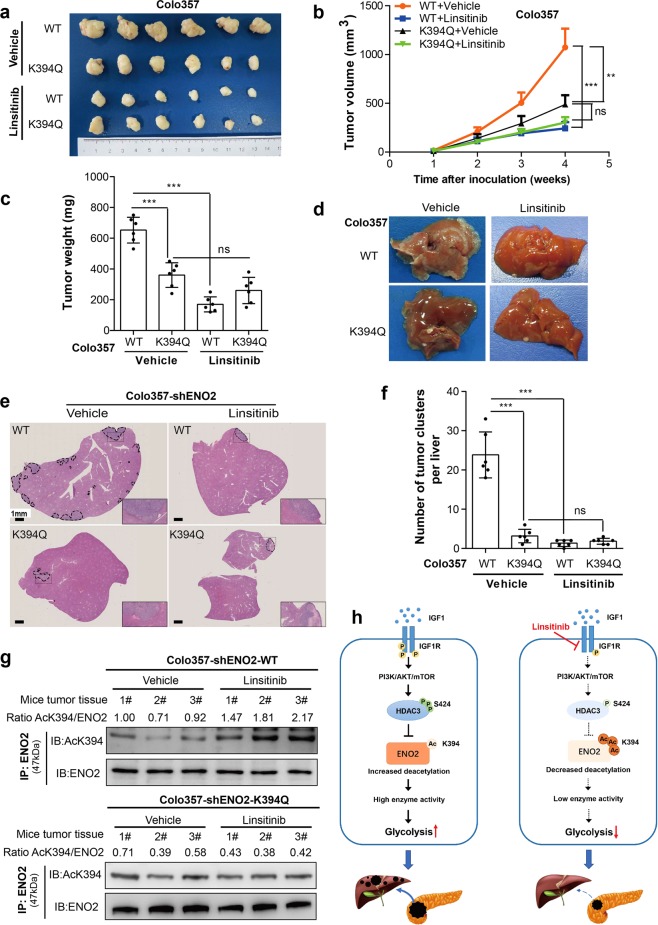
Inhibition of IGF-1R protects PDAC cells from IGF-1-induced tumor growth and metastasis. **a–c** In subcutaneous xenograft mouse models, Colo357 cells with ENO2 knockdown stably expressing wild-type ENO2 or K394Q mutants were subcutaneously injected into nude mice (*n* = 6 per group). When the tumor reached 100 mm^3^, the mice were treated with either 50 mg/kg linsitinib or vehicle once daily on a 5 days on and 3 days off cycle by oral gavage. Tumor volume (**a**), tumor growth curves (**b**) and tumor weight (**c**) were analyzed. **d–f** In metastatic mouse models, Colo357 cells (2.5 × 10^5^ cells per mouse, *n* = 6 per group) as indicated were intrasplenically injected, and mice were subsequently treated with either 50 mg/kg linsitinib or vehicle once daily on a 5 days on and 3 days off cycle by oral gavage. Metastatic nodule detection (**d**), H&E staining (**e**) and analysis of metastatic lesions (**f**) in the livers were performed (scale bar = 1 mm). **g** Xenograft tumor tissues as indicated were lysed and subjected to IP and western blot with anti-ENO2 and anti-AcK394 antibodies. K394 acetylation levels were normalized against ENO2 protein levels. **h** A working model showing that ENO2 K394 deacetylation plays a key role in promoting the metastasis of PDAC. Enolase 2 (ENO2), a key glycolytic enzyme, is acetylated by PCAF and deacetylated by HDAC3 at K394. The IGF-1/PI3K/Akt/mTOR pathway induces K394 deacetylation and stimulates ENO2 activity by increasing the phosphorylation of HDAC3 at S424, thereby promoting the growth and metastasis of PDAC. Linsitinib, an oral small-molecule inhibitor of IGF-1R, could inhibit IGF-1-induced ENO2 deacetylation and provide a promising strategy to prevent the development and progression of PDAC. Error bars represent the mean ± SD, and the dots represent the value of each experiment; **P < 0.01, ***P < 0.001, ns: no significance. Two-way ANOVA followed by Bonferroni’s post hoc test was employed in (**b**), and one-way ANOVA followed by Bonferroni’s post hoc test was employed in (**c**) and (**f**)

To confirm whether the IGF-1/mTOR pathway regulates K394 deacetylation and the activity of ENO2, we tested the effects of IGF-1R inhibition on the growth and metastasis of PDAC cells driven by overexpression of WT or K394-mutant ENO2 in vivo. Colo357 cells with ENO2 knockdown stably overexpressing WT or K394-mutant ENO2 were used to establish a subcutaneous implantation mouse model and metastatic mouse model, respectively. In subcutaneous xenograft model, treatment of mice with linsitinib was found to decrease the growth rates and sizes of tumors originating from Colo357 cells overexpressing WT ENO2 but had no effect on the growth rates and sizes of tumors derived from cells expressing K394-mutant ENO2 (Fig. 6a–c). In addition, in the metastatic mouse model, linsitinib treatment dramatically decreased liver metastasis of tumors originating from Colo357 cells overexpressing WT ENO2 but not that of tumors originating from cells overexpressing K394-mutant ENO2 (Fig. 6d–f). Furthermore, kidney and liver function studies in treated mice did not show any significant differences among the different treatment groups, suggesting no significant toxicity of the drug in either mouse model (Supplementary Fig. S8a, b). In addition, linsitinib treatment increased ENO2 K394 acetylation levels in mouse tumors originating from Colo357 cells overexpressing WT ENO2 but had no effect on ENO2 K394 acetylation in tumors derived from cells expressing K394-mutant ENO2 (Fig. 6g). Furthermore, treatment of mice with linsitinib significantly decreased the activation of the PI3K/AKT/mTOR pathway and HDAC3 S424 phosphorylation (Supplementary Fig. S8c).

As epithelial-mesenchymal transition (EMT) is suggested to be closely associated with cancer metastasis,^28^ we wondered whether EMT is the molecular mechanism by which IGF promotes PDAC metastasis by enhancing ENO2 activity. As expected, knockdown of ENO2-induced an increase in E-cadherin levels and significant decreases in the expression levels of N-cadherin, vimentin, Snail1 and Twist1 in both PDAC cells (Supplementary Fig. S9a) and subcutaneous xenograft models (Supplementary Fig. S9b). In addition, to identify whether IGF-1 is involved in the ENO2-induced enhancement of EMT, we assessed EMT markers in ENO2-upregulated PDAC cells treated with IGF-1 or linsitinib. Treatment with IGF-1 increased the EMT phenotype in ENO2-upregulated PDAC cells, with decreased E-cadherin and increased N-cadherin, vimentin, Snail1 and Twist1 levels. In contrast, linsitinib treatment led to a reversal of the pro-EMT effect by ENO2 both in vitro (Supplementary Fig. S9c) and in vivo (Supplementary Fig. S9d).

Together, these findings suggest that linsitinib treatment showed different effects on the growth and metastasis of PDAC cells depending on the cells overexpressed WT or K394-mutant ENO2. K394 acetylation plays a key role in regulating ENO2 function upon linsitinib treatment (Fig. 6h).

## Discussion

Metabolic reprogramming has been proven to play important roles in cancers.^5^ In pancreatic cancer, the extreme hypovascular and hypoxic microenvironments severely restrict nutrient availability. The adaptation of cellular metabolism by the tumor is particularly important for maximizing energy acquisition.^7^ ENO2, also referred to as NSE, is an important enzyme in glycolysis and a well-established tumor biomarker for small-cell lung cancer, prostate cancer, and so on.^15^ Studies have found that ENO2 is essential for cancer cell survival, proliferation and progression in glioblastoma multiforme and HCC.^16,29^ However, the function of ENO2 in pancreatic cancer has not been fully clarified. In our study, we first observed that high ENO2 expression was closely correlated with metastasis and poor survival in PDAC patients. Many recent works from our group and others have suggested that metabolic enzymes play important roles in EMT.^30–32^ Our current study reinforces the notion that metabolic enzymes such as ENO2 are an important promoter of PDAC metastasis, and more importantly, we found that ENO2 promotes PDAC metastasis by, at least in part, inducing EMT in PDAC cells. Given that PDACs are known to be highly malignant human tumors, it appears that ENO2 may serve as a novel metabolic biomarker for prognosis prediction, as well as a potential therapeutic target to block PDAC metastasis.

Previous studies have indicated that the majority of intermediate metabolic enzymes are acetylated and that the acetylation of metabolic enzymes plays an important role in tumorigenesis and the progression and metastasis of cancer.^11,13^ In our study, we determined that acetylation at K394, a site located in the active center of ENO2, played an important role in regulating ENO2 enzymatic activity and glycolytic metabolism in PDAC. More importantly, from investigations in clinical samples and cell lines, we provided both in vitro and in vivo evidence suggesting a novel function of ENO2 K394 deacetylation in promoting PDAC growth and metastasis. This suggests that ENO2 K394 deacetylation is in line with other regulatory mechanisms identified for ENO2 and may play a dramatic role in PDAC. Furthermore, these findings stress that aberrant deacetylation of ENO2 not only represents a key event contributing to PDAC metastasis but also can be targeted for the treatment of tumors with ENO2 deacetylation.

It is widely accepted that the biological activity of Kac is regulated by both acetyltransferases and deacetylases.^24^ We identified that both the acetyltransferase PCAF and the deacetylase HDAC3 play key roles in controlling ENO2 K394 acetylation. HDAC3, a member of the histone deacetylase family, is important in both physiological processes, such as the regulation of major metabolic and energy utilization pathways,^33^ and the development of different cancers.^34^ Previous studies have described the phosphorylation of S424 as critical for HDAC3 activity.^20^ Supporting this notion, we showed in this study that another metabolic enzyme, ENO2, is a direct substrate of HDAC3 and that the ENO2 deacetylation mediated by S424 phosphorylation of HDAC3 plays an important role in regulating ENO2 activity/function to drive PDAC progression, possibly via EMT.

IGF-1 exerts its effect through autocrine, paracrine, and endocrine mechanisms.^35^ IGF-1 plays an important role in tumorigenesis, angiogenesis, proliferation, and metastasis in various cancers.^36,37^ Studies have proven that the IGF-1/mTOR signaling pathway can facilitate cancer cell survival.^38^ The PI3K/AKT/mTOR pathway satisfies the metabolic demands for cancer proliferation, invasion and metastasis by allowing the utilization of amino acid substrates and one-carbon units.^25^ We demonstrate that the PI3K/AKT/mTOR pathway regulates ENO2 K394 deacetylation upon IGF-1 stimulation. Given the poor prognosis of PDAC, novel means of intervention are urgently needed. Several potent and selective IGF-1R inhibitors or antagonists are now in different stages of clinical development, including phase I/randomized phase II (dalotuzumab) and phase Ib/II (ganitumab) trials in multiple solid tumors.^39,40^ Linsitinib, also named OSI-906, is an orally bioavailable selective small-molecule tyrosine kinase inhibitor of both human IGF-1R and insulin receptor (IR).^41^ Linsitinib has been proven to have antiproliferative effects in cell lines and xenograft models in a variety of cancers.^42,43^ In our study, we observed that linsitinib treatment blocked the IGF-1-induced deacetylation of ENO2 and suppressed EMT, thereby inhibiting tumor growth and liver metastasis, indicating the potential clinical value of linsitinib in preventing liver metastasis in patients with deacetylated ENO2.

## Conclusions

Our study indicated that deacetylation of ENO2 K394 promotes metastasis of PDAC. Both the acetyltransferase PCAF and the deacetylase HDAC3 play key roles in regulating ENO2 K394 acetylation. The IGF-1/PI3K/Akt/mTOR pathway induces K394 deacetylation to stimulate ENO2 activity by increasing the phosphorylation of HDAC3 at S424, thereby promoting liver metastasis in PDAC. IGF-1R inhibitors such as linsitinib might be exploited for therapeutic benefit as effective adjuvant anticancer treatments for PDAC patients with deacetylated ENO2. Here, we propose an interesting possibility to develop a potential therapeutic strategy to block ENO2 function and control PDAC metastasis.

## Materials and methods

### Datasets, samples and follow-up

Data from GSE28735 and GSE15471 were retrieved from the public GEO database (http://www.ncbi.nlm.nih.gov/geo). The mRNA expression of target genes was calculated as fragments per kilobase of transcript per million fragments mapped values compared with β-actin. A total of 284 PDAC patients were enrolled in this study. Paired tumor and adjacent nontumor pancreas tissues were collected from patients who had undergone surgical resection for PDAC at the author’s institute with signed informed consent forms. The tissue specimens were immediately frozen in liquid nitrogen or fixed in 10% formalin and paraffin embedded. None of these patients had received any antitumor treatment preoperatively. The study was approved by the Ethics Committee of Fudan University (2018-164). In all 284 patients, 271 cases were collected between January 2012 and December 2014 for TMA construction. The clinicopathological profiles of the TMA cohort patients are summarized in Supplementary Table S1. The follow-up was completed in December 2017 with a median follow-up of 11 months (range 1–63 months). During the follow-up period, patients were re-evaluated by routine blood tests, computed tomography or magnetic resonance imaging every 3 months. An additional five paired tissues and eight pancreatic cancer tissues with no metastasis (from MFPs) or liver metastasis (from LMPs) from patients were collected to detect ENO2 and ENO2 K394 acetylation levels.

### TMA and IHC

Tumors and corresponding adjacent nontumor tissues from PDAC patients were systematically assembled to generate TMA blocks. IHC staining was performed on TMA-derived slides using a two-step method. For antigen retrieval, the tissue slides were treated with 0.01 mol/L sodium citrate (pH 6.0) in a microwave oven for 10 min after deparaffination and rehydration. The sections were then incubated with anti-ENO2 antibodies as described above at 4 °C overnight and subsequently developed using the ChemMate Dako EnVision Detection Kit, Peroxidase/DAB, Rabbit/Mouse (DakoCytomation, Glostrup, Denmark). The slides were then counterstained with hematoxylin. For negative controls, all procedures were conducted with the primary antibody omitted. Image-Pro Plus v6.0 software (Media Cybernetics Inc., MD, USA) was used to assess the immunostaining by mean optical density, as previously described.^44^ The cutoff of ENO2 was defined as the mean of all values.

### IP and western blot

The indicated cells were lysed in ice-cold NP-40 buffer containing 50 mM Tris-HCl (pH 7.4), 150 mM NaCl, 0.5% NP-40 and protease inhibitor cocktail (Roche, Basel, Switzerland) with rotation at 4 °C for 1 h. Lysates were centrifuged at 12,000 *×* *g* for 15 min at 4 °C. The supernatant was incubated with M2 anti-Flag mAb agarose beads (Merck & Co Inc., NJ, USA) for 3–5 h at 4 °C or incubated with the indicated antibody for 1 h, followed by incubation with Protein A beads (Santa Cruz Biotechnology, Inc., CA, USA) for another 3 h at 4 °C. The beads were then washed three times with ice-cold NP-40 buffer, and the protein was eluted by using Flag peptides (Merck & Co Inc., NJ, USA) according to the manufacturer’s protocol, followed by denaturation with SDS loading buffer. Western blot was performed as described in a previous study.^45^ For the acetylation western blot, buffer containing 50 mM Tris (pH 7.5) with 10% (v/v) Tween-20 and 1% peptone (Amresco, WA, USA) was used for blocking, and 5% BSA was used to prepare primary and secondary antibodies.

Frozen human PDAC tissues were ground and lysed in ice-cold 0.5% NP-40 buffer containing 50 mM Tris-HCl (pH 7.4), 150 mM NaCl, and protease and phosphatase inhibitor cocktail, with rotation at 4 °C for 1.5 h. IP was performed with the anti-ENO2 antibody, followed by western blot with the antibodies indicated. Immunoblotting intensity was quantified by using Image Quant TL software (GE Healthcare, USA).

### Antibody and chemicals

Antibodies against the following molecules were used in this study: ENO2 (#8171), pan-acetylated lysine (#9441), Flag (#14793), β-actin (#4970), HDAC3 (#85057), HA (#3724), Myc (#2276), PCAF (#3305), phospho-HDAC3-S424 (#3815), p-IGF-1R/IR (#3021), IGF-1R (#9750), IR (#3025), phospho-p70 S6K (Thr389) (#9234), AKT (#4691), pAkt-Ser473 (#4060), pAkt-Thr308 (#13038), ERK (#4695), pErk1/2 (Thr202/Tyr204) (#9101), vimentin (#5741), N-cadherin (#13116), Snail (#3879), E-cadherin (#3195), Twist1 (#46702) and ENO1 (#3810), which were purchased from Cell Signaling Technology (Danvers, MA, USA). The anti-ENO2 antibody (ab79757) was purchased from Abcam. A site-specific antibody to detect ENO2 K394 acetylation (anti-AcK394, 1:1000) was generated by synthesizing the peptide CTGQIK (Ac) TGAPCRS (Huiou Biotech Ltd., Shanghai, China), followed by coupling to KLH as an antigen to immunize rabbits. Antiserum was collected after four doses of immunization, followed by antibody purification.

Nicotinamide was purchased from Sigma-Aldrich. Trichostatin A, LY294002, rapamycin and linsitinib were purchased from Selleck Chemicals (Houston, TX, USA). Insulin, IGF-1, epidermal growth factor (EGF), transforming growth factor β (TGFβ), colony-stimulating factor (CSF1) and hepatocyte growth factor (HGF) were purchased from PeproTech (Rocky Hill, NJ, USA). Insulin (100 ng/mL, 12 h), IGF-1 (100 ng/mL, 12 h), EGF (30 ng/mL, 24 h), TGFβ (10 ng/mL, 72 h), CSF1 (10 ng/mL, 30 min) and HGF (50 ng/mL, 30 min) were used to treat PDAC cells for the indicated times. Biotinylated peptides of ENO2 containing either an acetylated or a nonacetylated K394 residue were purchased from Huiou Biotech Ltd (Shanghai, China).

### Enzyme activity

Flag-tagged ENO2 protein was overexpressed in HEK293T or Colo357 cells as indicated, followed by IP. The immunoprecipitate was eluted by the addition of 250 mg/mL Flag peptides dissolved in PBS (pH 7.5). ENO2 activity was measured using a fluorometric NADH-linked assay as previously described.^29^ The reactions were started by adding enzyme into the buffer containing 100 mM triethanolamine at pH 7.4, 56 mM DPG, 400 μM NADH, 5 mM MgSO4, 10 mM KCl, 2 mM ADP, and PK/LDH enzyme solution (Merck & Co Inc., NJ, USA). Enolase converts 2-PGA to PEP, the substrate of pyruvate kinase. Pyruvate formed by this reaction is linked to NADH oxidation by LDH. Enolase activity was determined by fluorescence measurement of NADH oxidation (Ex340 nm, Em460 nm) using a Hitachi F-4600 fluorescence spectrophotometer.

### ECAR and lactate assay

The ECAR assay was performed using a Seahorse Bioscience XFe 96 analyzer (Seahorse Bioscience, MA, USA) according to the manufacturer’s instructions. PDAC cells were counted and seeded into a Seahorse 96-well plate at a density of 1–2 × 10^4^ cells/well, followed by culture for 12 h. After that, 10 mM glucose, 2 μM oligomycin and 2-deoxy-D-glucose were added into different ports of the Seahorse cartridge. Each data point was the average of five independent measurements.

Lactate production of PDAC cells was quantified using the BioVision Lactate Assay Kit (Bio Vision, CA, USA). Then, 0.5 μL culture medium collected from the treated cells as indicated was added to lactate assay buffer. The reaction was incubated for 30 min at room temperature, and absorbance was measured at 570 nm using a microplate reader.

### Animal studies

All animal experiments were approved by the Animal Ethics Committee of Fudan University (201802088S). Colo357 cells with ENO2 knockdown and Colo357 cells with ENO2 knockdown plus reintroduced with WT ENO2 or K394Q mutants were injected subcutaneously into the flanks of 4-week-old male nude mice (5 × 10^6^ cells/mouse, *n* = 5–6 for each group). For treatment, when the tumor volume reached ~100 mm^3^, the mice were administered either 50 mg/kg linsitinib or vehicle orally once daily, on a 5 days on and 3 days off cycle, by oral gavage. Tumor volumes were measured every 4 days, and mice were sacrificed 28–32 days after injection. Xenograft tumors were collected and photographed, and tumor volume and weight were measured. In the metastatic NOD/SCID mouse model, Colo357 cells (2.5 × 10^5^ cells per mouse, *n* = 6) were implanted into the subcapsular region of the spleen of mice (5-week-old male NOD/SCID mice) as described previously.^46^ For treatment, mice were treated orally with either 50 mg/kg linsitinib or vehicle once daily, on a 5 days on and 3 days off cycle, by oral gavage 1 week after implantation and sacrificed 28 days later. The liver was harvested, and H&E staining was performed for metastatic nodule analysis.

### Statistical analysis

Quantitative data were expressed as the mean ± standard deviation (SD), while qualitative data were shown as the number (percent) or median (range) when appropriate. If the data were normally distributed and the variation between groups was minimal, paired Student’s *t* test was used to compare mean values between paired samples of two independent groups, and unpaired Student’s *t* test was applied for mean value comparison between two independent groups. If the variation among three or more groups was minimal, ANOVA followed by Bonferroni’s post hoc test was applied for comparison of multiple groups. Chi-square or Fisher’s exact test was used to determine whether there were any differences between two categorical variables. OS and PRR were compared by Kaplan-Meier curves, and the corresponding significance was determined using the log-rank test. The Cox regression model was applied to explore the effects of enrolled candidate variables. All statistical analyses were performed with SPSS version 13.0 (SPSS Inc., Chicago, USA). A two-sided *P* < 0.05 was considered statistically significant.

## Supplementary information

Supplementary material
