# Supplementary material for: Insulin-like growth factor 1-induced enolase 2 deacetylation by HDAC3 promotes metastasis of pancreatic cancer
Source: Signal Transduct Target Ther. 2020 May 13;5:53. doi: 10.1038/s41392-020-0146-6 (PMC7217878; doi:10.1038/s41392-020-0146-6)
Supplement: Supplementary file 1 — Supplementary material [file 41392_2020_146_MOESM1_ESM.docx]

**Supplementary Materials for**

**Insulin-like growth factor 1 induced enolase 2 de-acetylation by HDAC3 promotes metastasis of pancreatic cancer**

Yan Zheng^1^, Chao Wu^1^, Jimeng Yang^1^, Yue Zhao^2^, Huliang Jia^1^, Min Xue^1^, Da Xu^1^, Feng Yang^3^, Deliang Fu^3^, Chaoqun Wang^1^, Beiyuan Hu^1^, Ze Zhang^1^, Tianen Li^1^, Shican Yan^1^, Xuan Wang^1^, Peter J. Nelson^4^, Christiane Bruns^2^, Lunziu Qin^1^ and Qiongzhu Dong^1^

^1^Department of General Surgery, Huashan Hospital & Cancer Metastasis Institute & Institutes of Biomedical Sciences, Fudan University, Shanghai 200040, China; ^2^General, Visceral and Cancer Surgery, University Hospital of Cologne, Cologne, 50937, Germany; ^3^Department of Pancreatic Surgery, Pancreatic Disease Institute, Huashan Hospital, Shanghai Medical College, Fudan University, Shanghai, China and ^4^Medizinische Klinik und Poliklinik IV, Ludwig-Maximilian-University (LMU), Munich, Germany

Correspondence: Qiongzhu Dong ([qzhdong@fudan.edu.cn](mailto:qiongzhudong@163.com)) or Lunxiu Qin ([qinlx@fudan.edu.cn](mailto:qinlx@fudan.edu.cn))

These authors are considered equal first authors: Yan Zheng, Chao Wu, Jimeng Yang

**This PDF file includes:**

- MATERIALS AND METHODS
- FIGURES. S1 TO S9
- TABLES S1 TO S3

**MATERIALS AND METHODS**

Cell culture, plasmids and transfection

The human pancreatic cancer cell lines Colo357 and L3.6pl were provided by Prof. Christiane J. Bruns (General, Visceral and Cancer Surgery, University Hospital of Cologne, Germany). The human pancreatic duct epithelial (HPDE) cells and pancreatic cancer cell line FGF8 were provided by Prof. Kuizhi Wang (Southwest Hospital, Third Military Medical University, Chongqing, China). SW1990, PANC-1, Miacapa-2 and HEK293T cells were obtained from the Cell Bank of Shanghai Institutes of Biological Sciences, Chinese Academy of Sciences. L3.6pl Cells were cultured in Dulbecco’s modified Eagle’s medium (Gibco, 31885-023), with a 5% CO_2_ concentration at 37°C, supplemented with 12% FBS (Gibco,10099-141), 2% GlutaMAX (Gibco, 35050-061), 2% MEM Vitamin Solution (Gibco, 11120052), 2% MEM Non-Essential Amino Acids Solution (Gibco, 11140050), 20 U/mL penicillin and 20 μg/mL streptomycin (Sigma-Aldrich), and were trypsinised with 0.1% Trypsin (Gibco, 25200072) and passaged twice or three times per week. HPDE, SW1990, PANC-1, Miacapa-2, Colo357, FGF8 and HEK293T cells were cultured in Dulbecco’s modified Eagle’s medium (Gibco, 31885-023) with a 5% CO_2_ concentration at 37°C, supplemented with 10% FBS (Gibco, 10099-141), 20 U/mL penicillin and 20 μg/mL streptomycin (Sigma-Aldrich), and were trypsinised with 0.1% Trypsin (Gibco, 25200072) and passaged twice or three times per week.

The cDNA encoding full-length human ENO2 was cloned into Flag-tagged pCDH-CMV-MCS-EF1-Puro (CD510B-1, System Biosciences) using standard protocols. ENO2 K394Q/R point mutation was generated by Quik-Change Site-Directed Mutagenesis kit (Stratagene).

HA-tagged TIP60, HA-tagged HDACs (1-7), HA-HDAC3-S424A/D and Myc-tagged GCN5/PCAF (pcDNA3-HA; pcDNA3-Myc) were gifts from professor Kunliang Guan and Yue Xiong’s lab (Fudan University, Shanghai, China). HA-tagged hMOF/ CBP/ P300 were kind gifts from Professor Hongquan Zhang (Peking University, Peking, China). The shRNA sequences for ENO2, PCAF and HDAC3 were purchased from Sigma-Aldrich and cloned into pLKO.1 TRC (Addgene plasmid 10879). A scrambled siRNA precursor (Scr) was used as negative control. DNA sequencing were performed to verify the constructions. SiRNAs targeting GCN5/ PCAF/ TIP60/ hMOF/ CBP/ P300 were generated and validated as described in a previous study. ^1^ Sequences of shRNAs and siRNAs used in this study are listed in Supplementary Table S3. Transfection of Plasmids and siRNAs was performed by using lipofectamine 2000 (Invitrogen) as described in previous study. ^2, 3^

Colony formation assay

PDAC cells were seeded at a density of 800-1000 per well in six-well plates and treated with different doses of Linsitinib (2.5-10 μl) or dimethyl sulfoxide vehicle. Cells were cultured for 14 days, and then fixed and stained with crystal violet. Only colonies (clusters of more than 50 cells) visible to the naked eye were counted.

In vitro invasion assay

The in vitro invasion assay of the Colo357 and SW1990 cells as indicated were conducted using 24-well transwell chambers coated with matrigel (BD, San Jose, USA). Chambers have upper and lower culture compartments that are separated by polycarbonate membranes with 8μm pores (Costar, Cambridge, USA). The bottom chamber was filled with DMEM containing 10% FBS as a chemoattractant. Cells in serum-free medium were seeded at 2.5×10^4^ in the top chamber and incubated at 37°C in a humidified incubator containing 5% CO_2_. Cells that migrated to the underside of the membrane were stained with Giemsa solution (Sigma), imaged, and counted with a microscope (Leica, Wetzlar, Germany).

**REFERENCES**

1. Wang, S. et al. Insulin and mTOR Pathway Regulate HDAC3-Mediated Deacetylation and Activation of PGK1. *PLoS Biol.* **13**, e1002243 (2015).

2. Wang, Y. P. et al. Regulation of G6PD acetylation by SIRT2 and KAT9 modulates NADPH homeostasis and cell survival during oxidative stress. *EMBO J.* **33**, 1304-1320 (2014).

3. Ye, Q. H. et al. GOLM1 Modulates EGFR/RTK Cell-Surface Recycling to Drive Hepatocellular Carcinoma Metastasis. *Cancer Cell* **30**, 444-458 (2016).

**FIGURES. S1 TO S9**

**FIGURE S1**

**
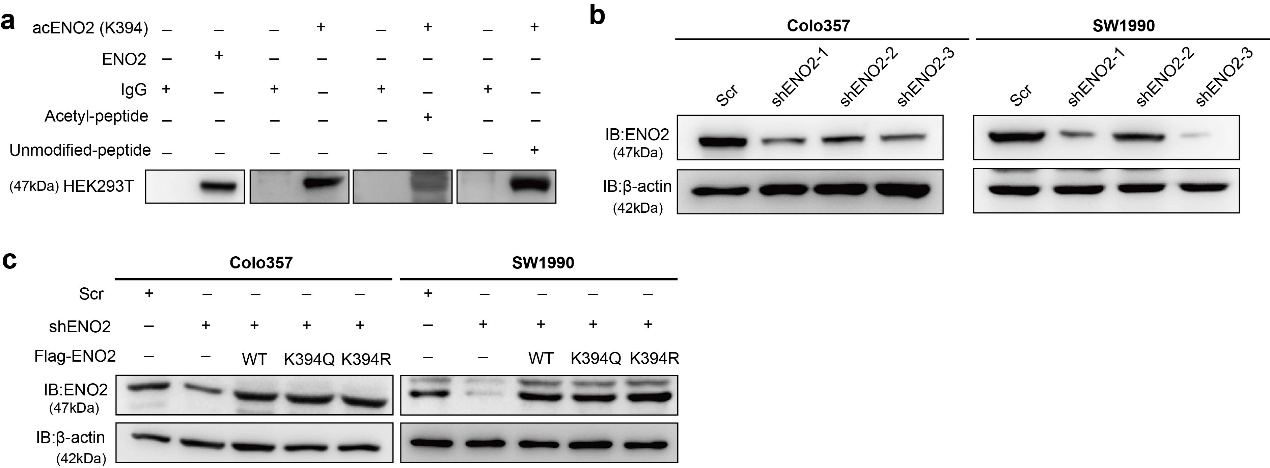
**

**Figure S1.** Validated the specificity of anti-acetyl-ENO2 K394 antibody and constructed stable cell lines. **a** HEK293T cell lysates were used for western blot with the IgG, ENO2 antibody, anti-acetyl-ENO2 K394 antibody, anti-acetyl-ENO2 K394 antibody incubating with acetylated peptide or unmodified peptide. **b** Knockdown of ENO2 in Colo357 and SW1990 cells were determined by western blot. **c** ENO2 was knocked down in Colo357 and SW1990 cells. Then wild type ENO2, K394R or K394Q mutants were re-introduced. Knockdown and re-expression efficiencies were determined by western blot.

**FIGURE S2**


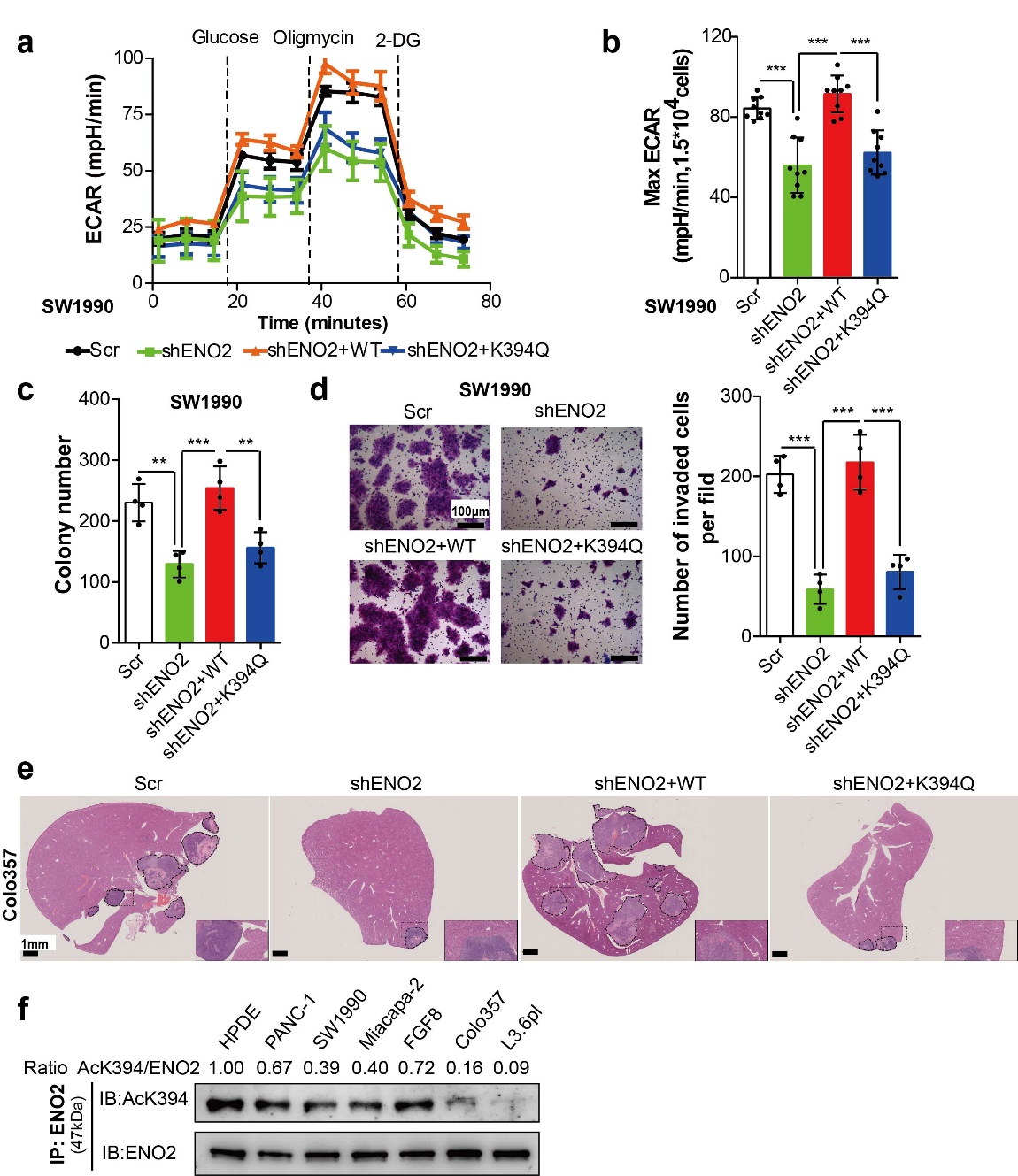


**Figure S2.** ENO2 K394 acetylation regulates PDAC glycolysis and metastasis. **a-b** ECAR assay was performed in SW1990 cells with Scr, shENO2 and reintroduction of ENO2 WT or K394Q mutant (**a**) and the max glycolytic rates were summarized (**b**, n = 9 per group). **c** Colony assays were performed with SW1990 cells indicated (n = 4 per group). **d** Transwell assays were used to detect the invasion ability of SW1990 cells indicated (n = 4 per group). **e** H&E staining were performed to count the number of liver metastatic nodules. **f** ENO2 K394 de-acetylation level was increased in L3.6pl cells, which showed high potential of liver metastasis. The human PDAC cell lines and a non-transformed pancreatic cell line (HPDE) were subjected to IP by using ENO2 antibody and its ENO2 K394 acetylation levels were determined by western blot and normalized against ENO2 protein level after quantification. Error bars represent the mean ± SD and the dots represent the value of each experiment; ***P*<0.01, ****P*<0.001, ns, no significance. Statistical significance was determined by one-way ANOVA followed by Bonferroni’s post hoc test.

**FIGURE S3**


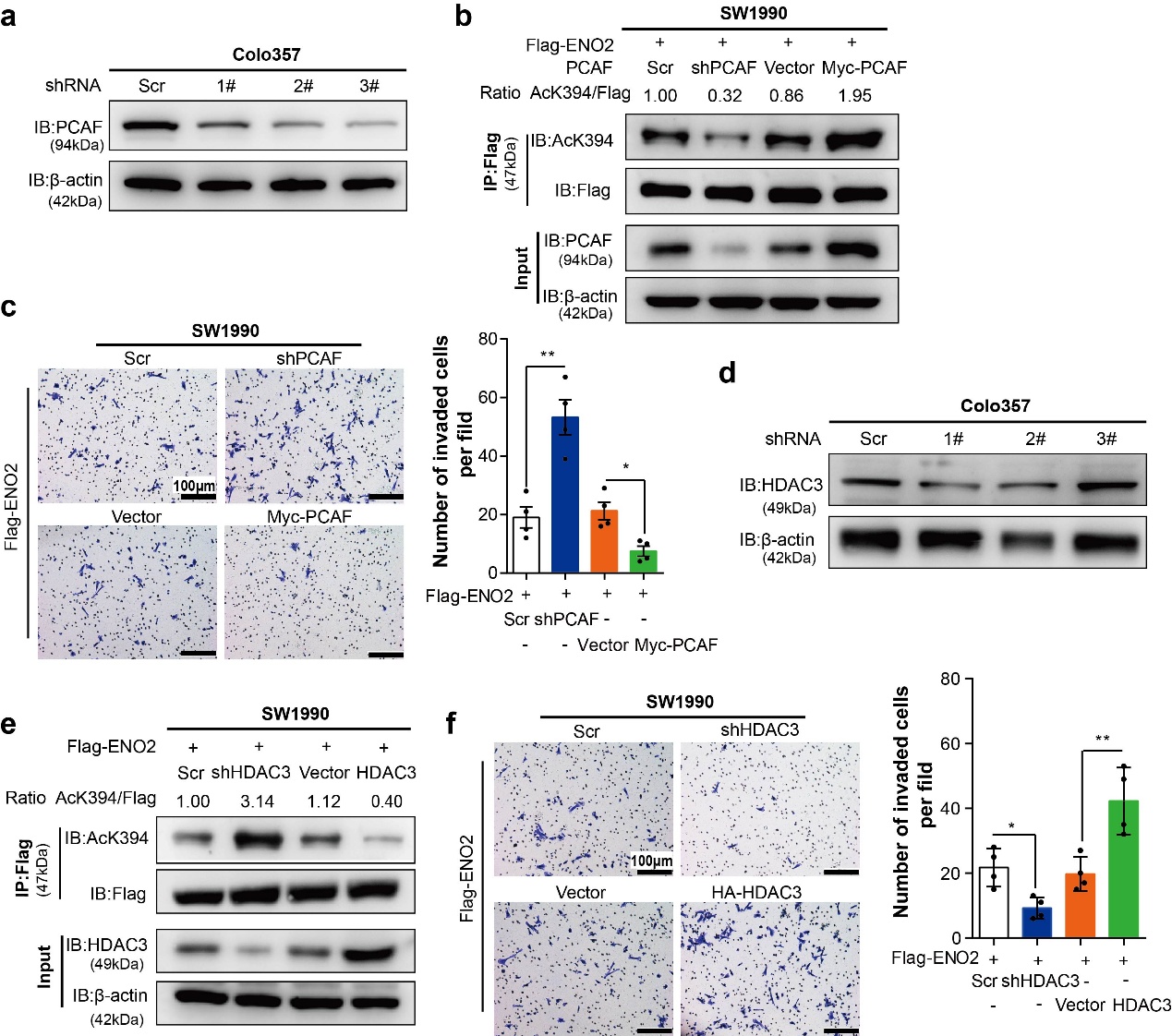


**Figure S3.** PCAF and HDAC3 are potential acetyltransferase and de-acetyltransferase of ENO2, respectively. **a** Depletion of PCAF expression in Colo357 cells with shRNAs and validation with western blot. **b-c** Knockdown or over-expression of Myc-tagged PCAF in SW1990 cells with ENO2 stably over-expressed, followed by TSA (5 μM) treatment for 12h. ENO2 K394 acetylation levels (**b**) and invasive ability (**c**, n = 4 per group) of SW1990 cells were determined. **d** Knockdown of HDAC3 were determined by western blot with anti-HDAC3 and anti-β-actin antibodies. **e-f** Knockdown or over-expression of HDAC3 in SW1990 cells with ENO2 stably over-expressed. ENO2 K394 acetylation levels were detected with anti-AcK394 antibody and normalized against Flag (**e**). Invasive ability of SW1990 cells indicated were determined by trans-well assays (**f**, n = 4 per group). Error bars represent the mean ± SD and the dots represent the value of each experiment; **P<0.05*, ***P*<0.01. Statistical significance was determined by unpaired *t* test.

**FIGURE S4**

**
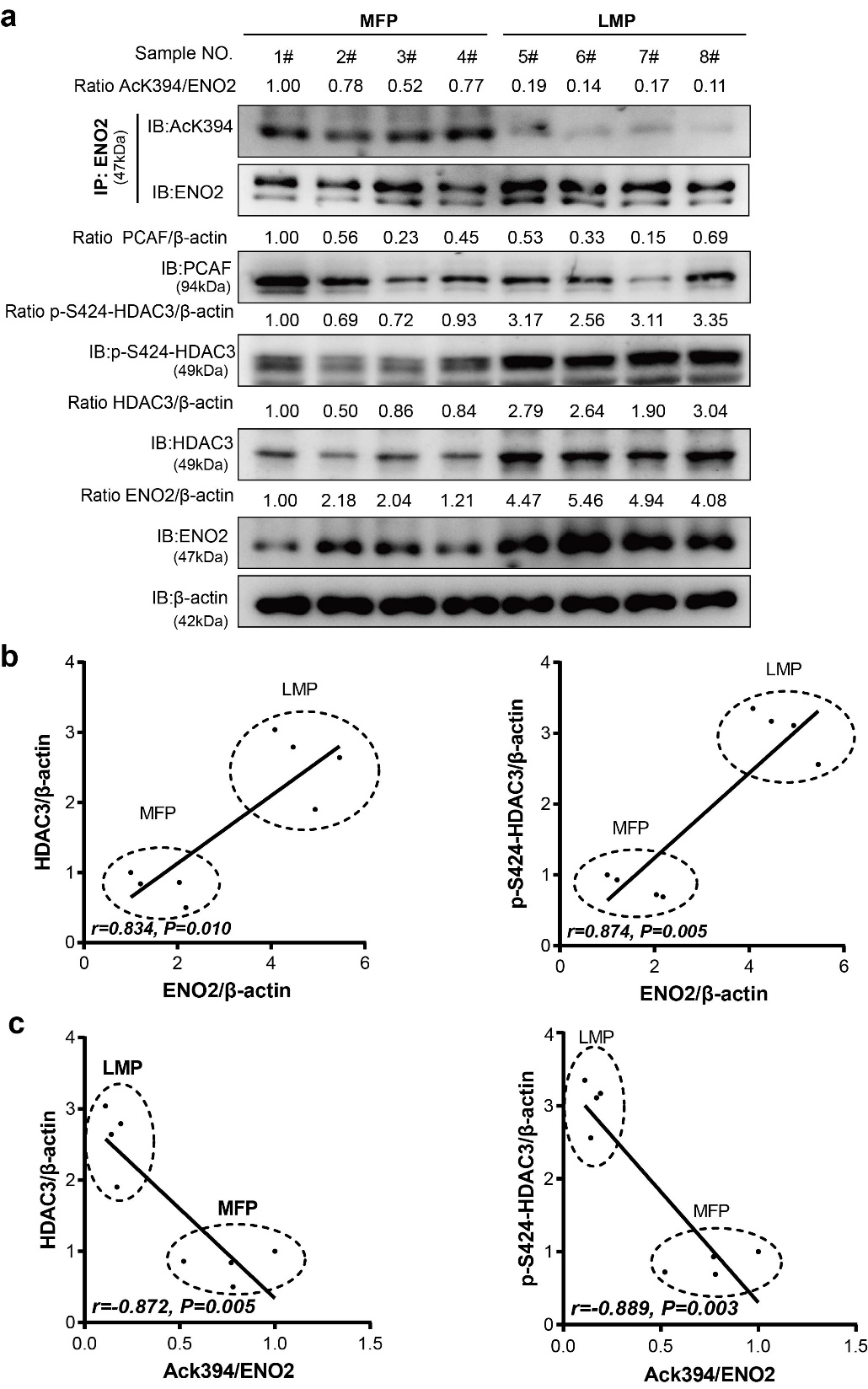
**

**Figure S4.** The expression level of HDAC3 and p-S424-HDAC3 in human PDAC tissues and their correlation with ENO2 K394 acetylation and ENO2 protein level. **a** The protein levels of ENO2, HDAC3, p-S424-HDAC38 and ENO2 K394 acetylation in 8 tumor samples with or without liver metastasis were determined by western blot with antibodies indicated. Relative protein levels were normalized against β-actin. MFP, metastasis free patients (n = 4); LMP, liver metastasis patients (n = 4). K394 acetylation levels were normalized against ENO2 protein level. **b-c** The correlation of HDAC3 and p-S424-HDAC3 protein levels with ENO2 protein level (**b**) and K394 acetylation levels (**c**). Statistical significance was determined by pearson correlation analysis.

**FIGURE S5**


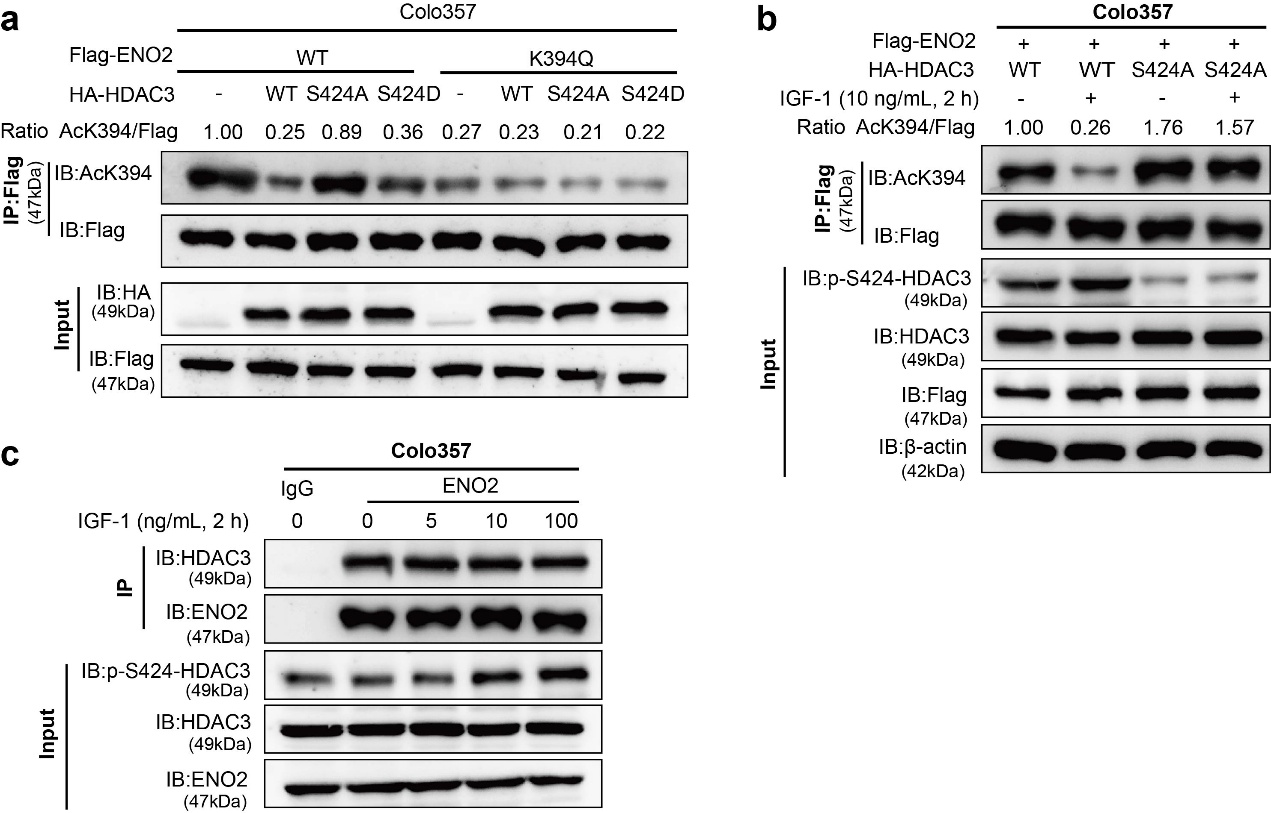


**Figure S5.** HDAC3 S424 is a key site for regulating HDAC3 mediating K394 de-acetylation of ENO2 upon IGF-1 stimulation. **a** Wild type ENO2 or ENO2 K394Q mutants were co-overexpressed with wild type HDAC3, HA-HDAC3 S424A mutant or S424D mutant in Colo357 cells. ENO2 was immunopurified with anti-Flag beads and its K394 acetylation was detected by western blot, normalizing against Flag. **b** Wild type ENO2 or ENO2 K394Q mutant were co-overexpressed with wild type HDAC3 or HA-HDAC3 S424A mutant in Colo357 cells following treatment with 0 or 10 ng/mL IGF-1 for 2 h. Immunoprecipitation and western blot were performed. K394 acetylation levels were normalized against Flag. **c** Colo357 cells extracts were immunoprecipitated with IgG or ENO2 antibody after simulated with different concentration of IGF-1 (0, 5, 10, 100 ng/mL) for 2 h. The protein level of HDAC3 and its Ser424 phosphorylation, and the association between endogenous HDAC3 and ENO2 were determined by western blot.

**FIGURE S6**

**
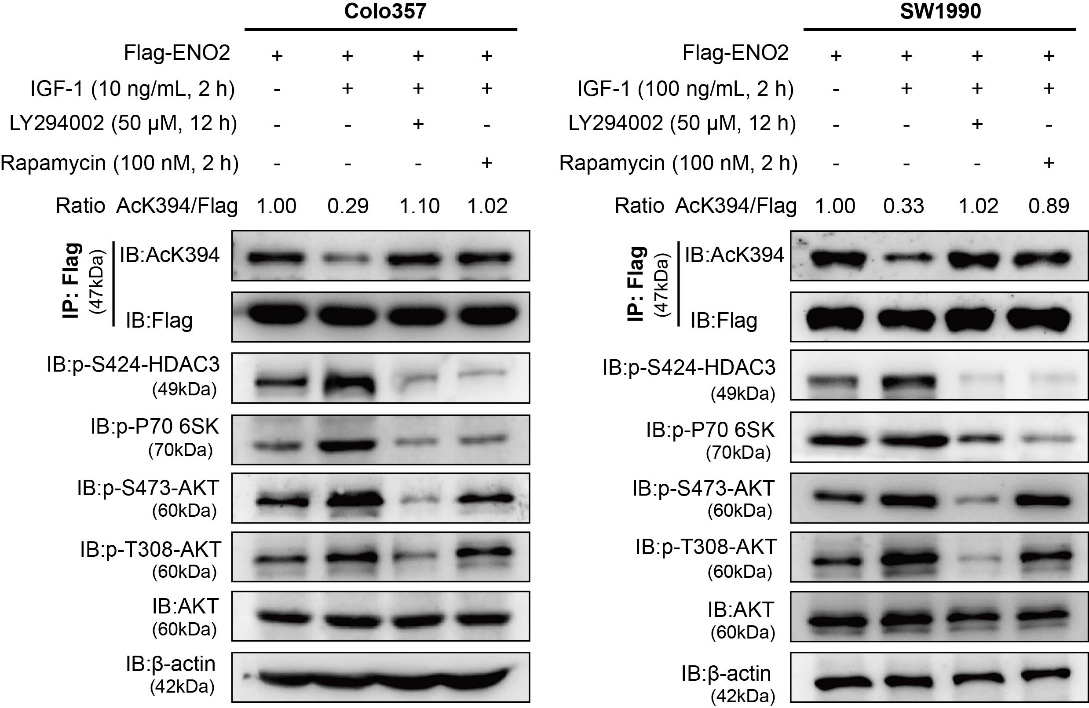
**

**Figure S6.** Inhibition of IGF-1/mTOR pathway could restrain the K394 de-acetylation induced by IGF-1. Colo357 and SW1990 cells were treated with LY294002 (PI3K inhibitor, 50μM for 12 h) or Rapamycin (mTOR inhibitor, 100nM for 2 h) after stimulation with IGF-1 (Colo357: 10 ng/mL and SW1990: 100 ng/mL) for 2h. Then cell lysates were subjected to immunoprecipitation and western blot with antibodies indicated to elucidate K394 acetylation levels (normalizing against Flag), the phosphorylation levels of HDAC3 S424 and activity of PI3K/AKT/mTOR pathway.

**FIGURE S7**


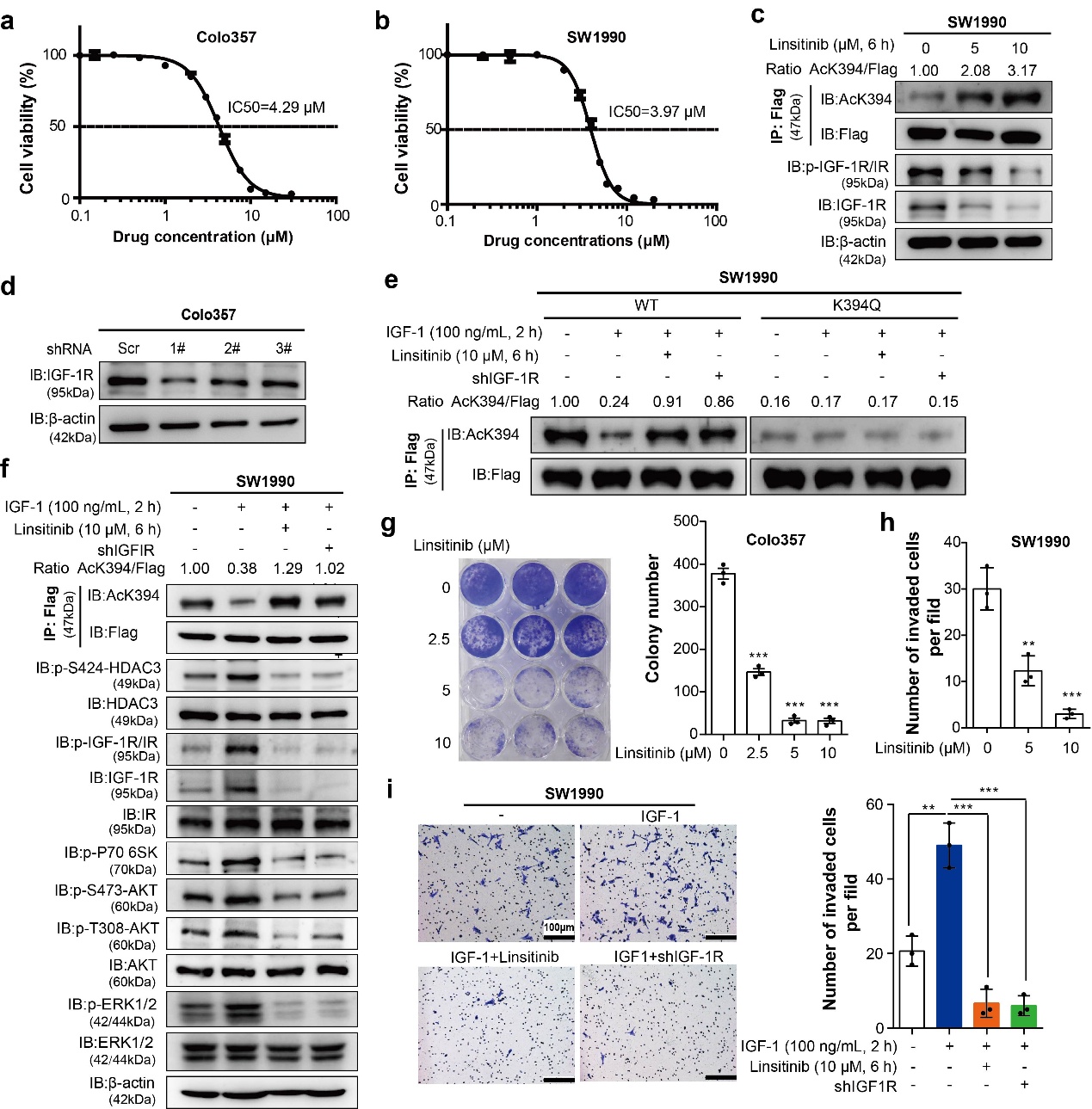


**Figure S7.** Linsitinib inhibits K394 de-acetylation of ENO2 regulated by IGF-1/mTOR pathway.

**a-b** Linsitinib, an insulin receptor/IGF-1R dual inhibitor, dose-dependently inhibited Colo357 cells (**a**, IC50=4.292 μM.) and SW1990 cells (**b**, IC50=3.970 μM.) viability after administration for 24 hours. **c** SW1990 cells stably over-expressing with Flag-tagged ENO2 were treated with 0, 5, 10 μM Linsitinib for 6 h. The phosphorylation of IGF-1R and K394 acetylation of ENO2 were determined by IP and western blot using antibodies indicated. **d** Western blot were performed to validate the knockdown of IGF-1R in Colo357 cells. **e** SW1990 cells ectopically expressed ENO2 were treated with IGF-1 (10 ng/mL, 2 h) stimulation or Linsitinib (10 μM, 6 h) plus IGF-1 (10 ng/mL, 2 h) stimulation or IGF-1R knockdown plus IGF-1 (10 ng/mL, 2 h) stimulation. ENO2 proteins were immunoprecipitated and K394 acetylation was examined with anti-AcK394, normalizing against Flag. **f** SW1990 cells treated as indicated, were subjected to immunoprecipitation and western blot with antibodies indicated to elucidate K394 acetylation levels (normalizing against Flag), the phosphorylation levels of HDAC3 S424 and activity of PI3K/AKT /mTOR and ERK pathway. **g-h** Colony assays (**g**) and transwell assay (**h**) were performed with PDAC cells following by treating with 0, 2.5, 5, 10 μM Linsitinib, n = 3 per group. **i** Transwell assay was performed with the SW1990 cells indicated in Figure S7f (n = 3 per group). Error bars represent the mean ± SD and the dots represent the value of each experiment; **P<0.05*, ***P*<0.01, *** *P*<0.001. Nonlinear fit was used in **a** and **b**, and one-way ANOVA followed by Bonferroni’s post hoc test was used in **g-i**.

**FIGURE S8**

**
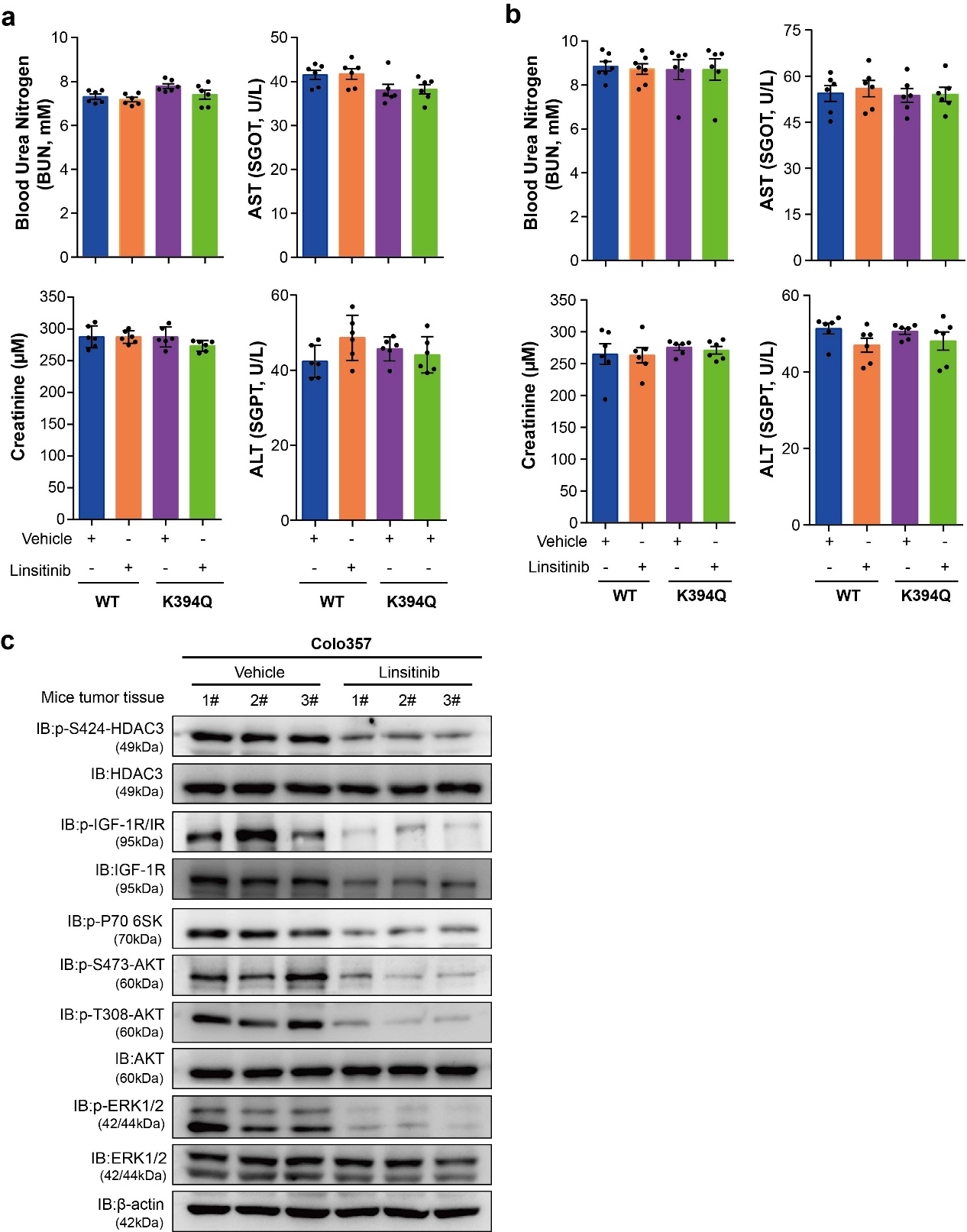
**

**Figure S8.** Effects of Linsitinib on liver and kidney function in mice model. **a** The effect of Linsitinib on liver and kidney function in subcutaneous xenograft mouse model. AST, aspartate aminotransferase. ALT, alanine transaminase, n = 6 per group. **b** The effect of Linsitinib on liver and kidney function in metastatic mouse model, n = 6 per group. **c** Xenograft tumor tissues from subcutaneous tumor model with or without Linsitinib treatment were lysed and subjected to western blot with antibodies indicated to elucidate the phosphorylation levels of HDAC3 S424 and activity of PI3K/AKT /mTOR pathway. Error bars represent the mean ± SD and the dots represent the value of each experiment.

**FIGURE S9**


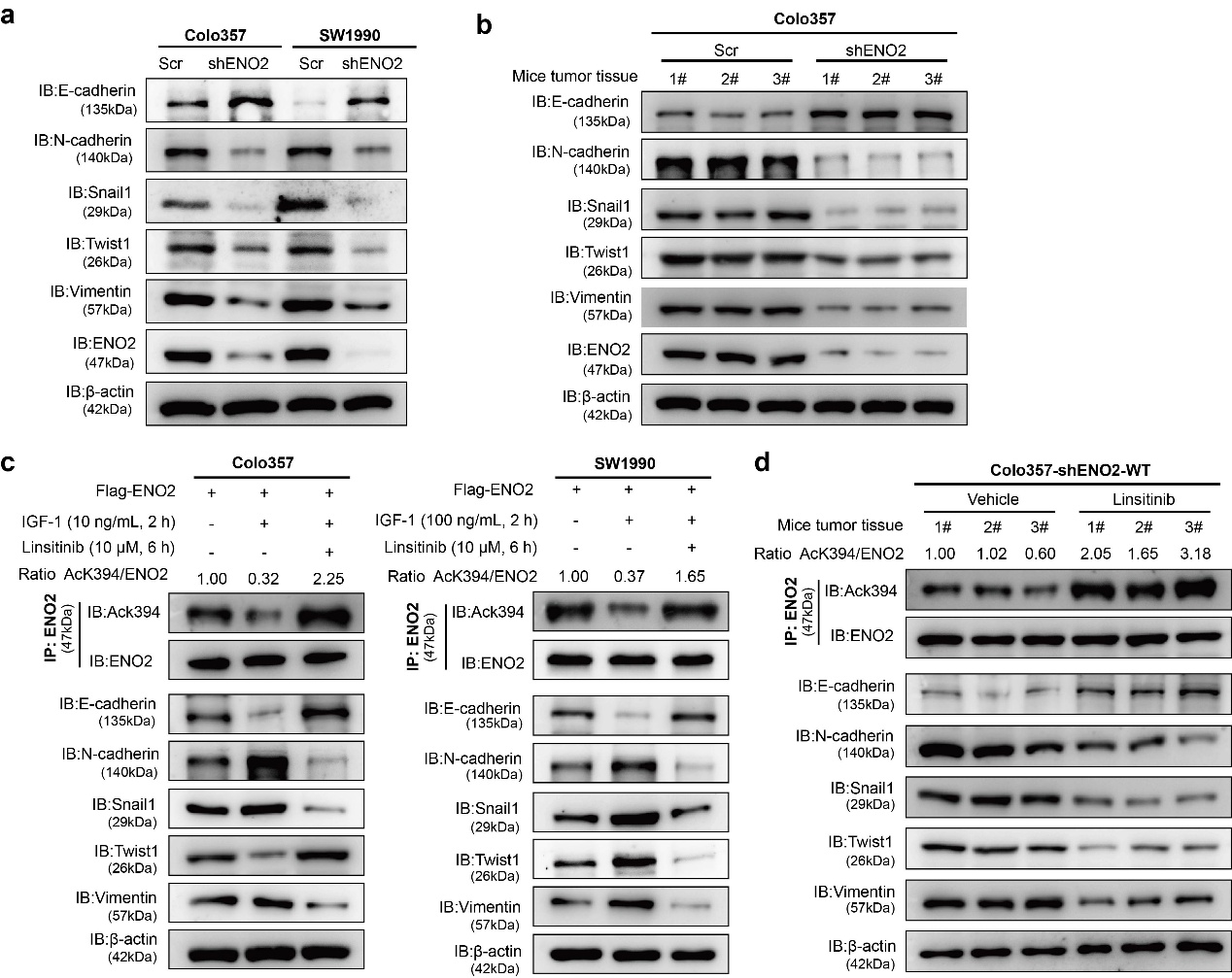


**Figure S9.** Knockdown of ENO2 or Linsitinib treatment inhibit EMT *in vitro* and *in vivo*. **a-b** Western blot were performed with Colo357 cells, SW1990 cells (**a**) and Xenograft tumors (**b**) in which ENO2 was knockdown, to analyze the protein levels of EMT markers. **c** PDAC cells with Flag-tagged ENO2 stably over-expressed were treated with Linsitinib (10 μM) for 6 h, after IGF-1 (10 ng/mL, 2 h for Colo357 cells and 100 ng/mL, 2 h for SW1990 cells) stimulation. ENO2 proteins were immunoprecipitated and K394 acetylation was examined with anti-AcK394, normalizing against Flag. EMT markers were detected by western blot with antibodies indicated. **d** Xenograft tumor tissues from subcutaneous tumor model treated with or without Linsitinib were subjected to immunocoprecipitation and western blot to determine the levels of EMT markers and K394 acetylation of ENO2 with antibodies indicated. K394 acetylation levels were normalized against ENO2 protein levels.

**TABLES S1 TO S3**

**TABLES S1.**

**TABLES S1.** Comparison of clinic-pathologic profiles between low and high ENO2 expression in pancreatic cancer patients (n=271)

| Variables | ENO2 Expression | | | | |
| --- | --- | --- | --- | --- | --- |
|  | Low(N=135) | | High(N=136) | | *P*^a^ |
|  | No. of patients | % | No. of patients | % |  |
| Gender |  |  |  |  |  |
| Female | 57 | 42.2% | 55 | 40.4% | 0.766 |
| Male | 78 | 57.8% | 81 | 59.6% |  |
| Age(years) |  |  |  |  |  |
| ＜60 | 66 | 48.9% | 51 | 37.5% | 0.058 |
| ≥60 | 69 | 51.1% | 85 | 62.5% |  |
| BMI(kg/m^2^) |  |  |  |  |  |
| ＜24 | 73 | 54.1% | 79 | 58.1% | 0.506 |
| ≥24 | 62 | 45.9% | 57 | 41.9% |  |
| Tbil (μmol/dl) | |  |  |  |  |
| ≤17.1 | 54 | 40.0% | 46 | 33.8% | 0.292 |
| ＞17.1 | 81 | 60.0% | 90 | 66.2% |  |
| CEA(U/ml) | |  |  |  |  |
| ≤10 | 123 | 91.1% | 122 | 89.7% | 0.695 |
| ＞10 | 12 | 8.9% | 14 | 10.3% |  |
| CA19-9(U/ml) |  |  |  |  |  |
| ≤37 | 54 | 40.0% | 41 | 30.1% | 0.089 |
| ＞37 | 81 | 60.0% | 95 | 69.9% |  |
| Tumor size(cm) | |  |  |  |  |
| ＜4 | 84 | 62.2% | 87 | 64.0% | 0.766 |
| ≥4 | 51 | 37.8% | 49 | 36.0% |  |
| Tumor differentiation | |  |  |  |  |
| Ⅰ+Ⅱ | 82 | 60.7% | 66 | 48.5% | 0.044 |
| Ⅲ+Ⅳ | 53 | 39.3% | 70 | 51.5% |  |
| pTNM stage |  |  |  |  |  |
| Ⅰ | 56 | 41.5% | 50 | 36.8% | 0.426 |
| Ⅱ | 79 | 58.5% | 86 | 63.2% |  |
| Positive lymph node status | | |  |  |  |
| No | 78 | 57.8% | 50 | 36.8% | <0.001 |
| Yes | 57 | 42.2% | 86 | 63.2% |  |

^a^ Chi-square test and *P<0.05* was considered statistically significant.

Abbreviations: BMI, body mass index; Tbil, total bilirubin; CEA, carcinoembryonic antigen; CA19-9, carbohydrate antigen 19-9; pTNM, pathological tumor, lymph node, metastasis classification.

**TABLES S2.**

**TABLES S2.** Univariate and multivariate analysis of factors associated with survival and recurrence in pancreatic cancer patients (n=271)

| Variables | Survival ^a^ | | | | | | |  | Recurrence ^a^ | | | | | | |
| --- | --- | --- | --- | --- | --- | --- | --- | --- | --- | --- | --- | --- | --- | --- | --- |
|  | univariate analysis | | |  | multivariate analysis | | |  | univariate analysis | | |  | multivariate analysis | | |
|  | HR | 95% CI | *P* value |  | HR | 95% CI | *P* value |  | HR | 95% CI | *P* value |  | HR | 95% CI | *P* value |
| Gender (male vs female) | 1.350 | 1.015-1.797 | 0.039 |  | 1.263 | 0.944-1.688 | NS |  | 1.284 | 0.976-1.689 | NS |  |  |  | NA |
| Age (≥60 vs ＜60) | 1.026 | 0.776-1.356 | NS |  |  |  | NA |  | 1.077 | 0.822-1.413 | NS |  |  |  | NA |
| BMI (≥24kg/m^2^ vs ＜24kg/m^2^) | 0.894 | 0.676-1.182 | NS |  |  |  | NA |  | 0.857 | 0.653-1.125 | NS |  |  |  | NA |
| Tbil (＞17.1μmol/dl vs ≤17.1μmol/dl) | 1.017 | 0.763-1.356 | NS |  |  |  | NA |  | 1.000 | 0.757-1.320 | NS |  |  |  | NA |
| CA19-9 (＞37U/ml vs ≤37U/ml) | 1.462 | 1.086-1.968 | 0.012 |  | 1.394 | 1.031-1.884 | 0.031 |  | 1.618 | 1.208-2.167 | 0.001 |  | 1.509 | 1.125-2.024 | 0.006 |
| CEA (＞10U/ml vs ≤10U/ml) | 0.955 | 0.588-1.550 | NS |  |  |  | NA |  | 0.900 | 0.562-1.442 | NS |  |  |  | NA |
| Tumor size (≥4cm vs ＜4cm) | 0.970 | 0.728-1.291 | NS |  |  |  | NA |  | 0.939 | 0.711-1.241 | NS |  |  |  | NA |
| Positive lymph node status(yes vs no) | 1.254 | 0.950-1.656 | NS |  |  |  | NA |  | 1.247 | 0.953-1.632 | NS |  |  |  | NA |
| Tumor differentiation (Ⅲ+ⅣvsⅠ+Ⅱ) | 2.251 | 1.695-2.990 | <0.001 |  | 2.030 | 1.520-2.713 | <0.001 |  | 2.277 | 1.727-3.001 | <0.001 |  | 2.099 | 1.587-2.775 | <0.001 |
| pTNM stage (ⅡvsⅠ) | 1.002 | 0.756-1.327 | NS |  |  |  | NA |  | 0.971 | 0.739-1.275 | NS |  |  |  | NA |
| ENO2 expression (high vs low) | 1.715 | 1.295-2.270 | <0.001 |  | 1.537 | 1.157-2.042 | 0.003 |  | 1.687 | 1.286-2.213 | <0.001 |  | 1.562 | 1.188-2.053 | 0.001 |

^a^ Cox proportional hazards regression

Abbreviations: BMI, body mass index; Tbil, total bilirubin; CEA, carcinoembryonic antigen; CA19-9, carbohydrate antigen 19-9; pTNM, pathological tumor, lymph node, metastasis classification; NA, not adopted; NS, not significant.

**TABLES S3.**

**TABLES S3.** The list of shRNA sequences used in this study

| Name | Sequence(5'-3') | Note |
| --- | --- | --- |
| shENO2#1 | CAAGGGAGTCATCAAGGACAA | shRNA |
| shENO2#2 | CGCCTGGCTAATAAGGCTTTA | shRNA |
| shENO2#3 | CATCAAGGACAAATACGGCAA | shRNA |
| shPCAF#1 | CGAACTCTAATCCTCACTCAT | shRNA |
| shPCAF#2 | GCAGACTTACAGCGAGTCTTT | shRNA |
| shPCAF#3 | GTTGGCTATATCAAGGATTAT | shRNA |
| shHDAC3#1 | CAAGAGTCTTAATGCCTTCAA | shRNA |
| shHDAC3#2 | CCTTCCACAAATACGGAAATT | shRNA |
| shHDAC3#3 | GATCTGTGATATTGCCATTAA | shRNA |
| shIGF-1R#1 | GCCTTTCACATTGTACCGCAT | shRNA |
| shIGF-1R#2 | GCCGAAGATTTCACAGTCAAA | shRNA |
| shIGF-1R#3 | CCAAGCCTGAGCAAGATGATT | shRNA |
